# PNPO–PLP axis senses prolonged hypoxia in macrophages by regulating lysosomal activity

**DOI:** 10.1038/s42255-024-01053-4

**Published:** 2024-05-31

**Authors:** Hiroki Sekine, Haruna Takeda, Norihiko Takeda, Akihiro Kishino, Hayato Anzawa, Takayuki Isagawa, Nao Ohta, Shohei Murakami, Hideya Iwaki, Nobufumi Kato, Shu Kimura, Zun Liu, Koichiro Kato, Fumiki Katsuoka, Masayuki Yamamoto, Fumihito Miura, Takashi Ito, Masatomo Takahashi, Yoshihiro Izumi, Hiroyuki Fujita, Hitoshi Yamagata, Takeshi Bamba, Takaaki Akaike, Norio Suzuki, Kengo Kinoshita, Hozumi Motohashi

**Affiliations:** 1 Department of Medical Biochemistry, Tohoku University Graduate School of Medicine, Sendai, Japan; 2 Division of Cardiology and Metabolism, Center for Molecular Medicine, Jichi Medical University, Shimotsuke, Japan; 3 Department of Cardiovascular Medicine, Graduate School of Medicine, The University of Tokyo, Tokyo, Japan; 4 Department of Gene Expression Regulation, IDAC, Tohoku University, Sendai, Japan; 5 Department of System Bioinformatics, Graduate School of Information Sciences, Tohoku University, Sendai, Japan; 6 Department of Integrative Genomics, Tohoku Medical Megabank Organization, Tohoku University, Sendai, Japan; 7 Data Science Center, Jichi Medical University, Shimotsuke, Japan; 8 Division of Oxygen Biology, United Centers for Advanced Research and Translational Medicine, Tohoku University Graduate School of Medicine, Sendai, Japan; 9 Department of Biochemistry and Molecular Biology, Tohoku Medical Megabank Organization, Tohoku University, Sendai, Japan; 10 Department of Biochemistry, Kyushu University Graduate School of Medical Sciences, Fukuoka, Japan; 11 Division of Metabolomics, Medical Institute of Bioregulation, Kyushu University, Fukuoka, Japan; 12 Advanced Research Laboratory, Canon Medical Systems Corporation, Otawara, Japan; 13 Department of Environmental Medicine and Molecular Toxicology, Tohoku University Graduate School of Medicine, Sendai, Japan

**Keywords:** Metabolomics, Lysosomes, Inflammation, Metabolism, Monocytes and macrophages

## Abstract

Oxygen is critical for all metazoan organisms on the earth and impacts various biological processes in physiological and pathological conditions. While oxygen-sensing systems inducing acute hypoxic responses, including the hypoxia-inducible factor pathway, have been identified, those operating in prolonged hypoxia remain to be elucidated. Here we show that pyridoxine 5′-phosphate oxidase (PNPO), which catalyses bioactivation of vitamin B6, serves as an oxygen sensor and regulates lysosomal activity in macrophages. Decreased PNPO activity under prolonged hypoxia reduced an active form of vitamin B6, pyridoxal 5′-phosphate (PLP), and inhibited lysosomal acidification, which in macrophages led to iron dysregulation, TET2 protein loss and delayed resolution of the inflammatory response. Among PLP-dependent metabolism, supersulfide synthesis was suppressed in prolonged hypoxia, resulting in the lysosomal inhibition and consequent proinflammatory phenotypes of macrophages. The PNPO–PLP axis creates a distinct layer of oxygen sensing that gradually shuts down PLP-dependent metabolism in response to prolonged oxygen deprivation.

## Abstract (web summary)

Sekine et al. show that macrophages utilize different pathways to sense acute and chronic hypoxia, the latter relying preferentially on PNPO–PLP rather than hypoxia-inducible factors.

## Main

Oxygenation of the Earth’s atmosphere resulted in the emergence of aerobic organisms that utilize molecular oxygen (O_2_) for energy metabolism in mitochondria and many other reactions catalysed by oxygenases and oxidases. Oxygenases incorporate oxygen atoms from O_2_ into their substrates, whereas oxidases use O_2_ as an electron acceptor. All metazoan organisms have been shown to possess oxygen-sensing mechanisms for adaptation to low-oxygen conditions^1^, which utilize dioxygenases with high Michaelis constant (*K*_m_) values for oxygen, namely PHD^2^, KDM6A^3^ and ADO^4^, as oxygen sensors; however, how oxidases make contributions to oxygen sensing and response to hypoxia is not fully understood.

Among the dioxygenases, PHD and its effector hypoxia-inducible factor (HIF) form a major molecular system that mediates the response to hypoxia^5^. PHD belongs to the 2-oxoglutarate (2OG)-dependent dioxygenase family and requires molecular oxygen as a substrate^6,7^. Because the *K*_m_ value of PHD for oxygen is high enough, PHD activity easily declines under hypoxia, resulting in the stabilization of HIFα subunits and activation of HIF target genes. Thus, a high *K*_m_ value for oxygen allows PHD to serve as an oxygen sensor. KDM6A has been shown to serve as another oxygen sensor, regulating the epigenetic status in response to hypoxia, because the *K*_m_ value of KDM6A for oxygen is similarly high to that of PHD^2,3^. In contrast, KDM5A and TET activities have been reported to be sensitive to oxygen tension, although their *K*_m_ values for O_2_ are rather low^3,8–10^, implying alternative oxygen-sensing mechanisms for the regulation of 2OG-dependent dioxygenase activities.

Crosstalk between hypoxia and inflammation is understood at the molecular level as a functional interaction between HIF-1α, a key regulator of the hypoxic response, and nuclear factor (NF)-κB, a key regulator of the inflammatory response^11^. HIF-1α has been shown to enhance interleukin (IL)-1β production and drive inflammation by inducing a metabolic shift to glycolysis from oxidative phosphorylation in activated macrophages^12^. Deleting HIF-1α in myeloid cells attenuates inflammation, verifying an important role of HIF-1α as a proinflammatory regulator in vivo^13^. In good agreement with the HIF-1α requirement in the inflammatory response of macrophages, acute hypoxia augments the inflammatory response by activating NF-κB signalling in vitro and in mice^11^. These studies provide solid evidence for the proinflammatory effects of acute hypoxia, which is mediated by HIF-1α; however, this mechanistic scheme seems less applicable to prolonged hypoxia because of the transient nature of HIF-1α-mediated transcriptional activation^11^. A question here is how the prolonged hypoxia is sensed, responded to and involved in the inflammatory processes.

Pyridoxal 5′-phosphate (PLP) is an active form of vitamin B6 and serves as a coenzyme for many amino acid-metabolizing enzymes. Pyridoxine is a major form of vitamin B6 in food and undergoes bioactivation to become PLP, which is catalysed by pyridoxine 5′-phosphate oxidase (PNPO), requiring molecular oxygen as a substrate. PLP-dependent enzymes include transaminase, decarboxylase and supersulfide-synthesizing enzymes, such as CBS, CSE, CASR1 and CARS2. Supersulfides are sulfur species with catenated sulfur atoms, including hydropersulfides (RSSHs) and polysulfide species and exist as low molecular weight metabolites and as proteins with excess sulfur atoms in the side chains of cysteine residues^14,15^. Among various physiological roles of supersulfides, their anti-inflammatory function has been well described^16–18^.

Here we demonstrate that PNPO serves as an oxygen sensor and inhibits lysosomal activity under prolonged hypoxia irrespective of the HIF pathway status. In macrophages, prolonged hypoxia, but not acute hypoxia, reduces lysosomal acidification, which limits ferrous iron (Fe^2+^) availability and switches off the TET2 function that mediates inflammatory resolution. Metabolome analysis revealed that prolonged hypoxia suppresses vitamin B6 bioactivation catalysed by PNPO, leading to a decrease in the production of supersulfides, which were found to be critical for maintaining lysosomal acidification. Supplementation of active vitamin B6 restored intracellular supersulfides, lysosomal acidification and TET2 protein accumulation in macrophages and attenuated inflammatory response in mice under prolonged hypoxia. This study has identified PNPO–PLP axis as an alternative oxygen-sensing system operative in prolonged hypoxia, which is distinct from dioxygenase-dependent mechanisms, including the PHD–HIF pathway.

## Results

### Prolonged hypoxia exacerbates inflammation

**Fig. 1:**
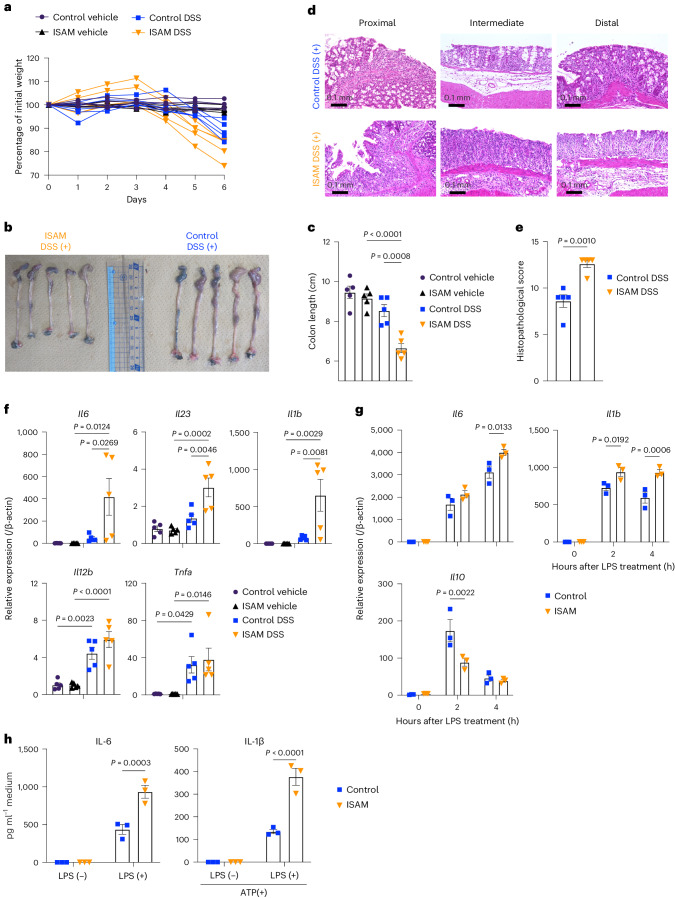
Prolonged hypoxia model mice ISAM (male) are highly susceptible to DSS-induced colitis. **a**, Body weight changes in ISAM and control mice during treatment with 3% DSS or vehicle (water) (*n* = 5 mice for each group). *P* < 0.0001 and *P* = 0.0001 for comparison of control DSS and ISAM DSS on day 5 and day 6, respectively. **b**–**e**, Colonic pathological changes on day 6 of treatment with 3% DSS. Macroscopic appearance (**b**), length (**c**), haematoxylin and eosin-stained sections (**d**) and histopathological scores (**e**) of colons in ISAM and control mice (*n* = 5 mice for each group). Scale bars, 100 μm (**d**). **f**,**g**, Expression of cytokine genes in the colons of ISAM and control mice with or without DSS treatment for 6 days (*n* = 5 mice for each group) (**f**) and in PMs from ISAM and control mice (*n* = 3 mice for each group) (**g**). **h**, ELISA of cytokines in the culture supernatant of PMs from ISAM and control mice (*n* = 3 mice for each group). Error bars represent s.e.m. A two-way ANOVA (**a**,**g**,**h**), one-way ANOVA (**c**,**f**) and two-sided Student’s *t*-test (**e**) were conducted to evaluate statistical significance. Source data

**Extended Data Fig. 1:**
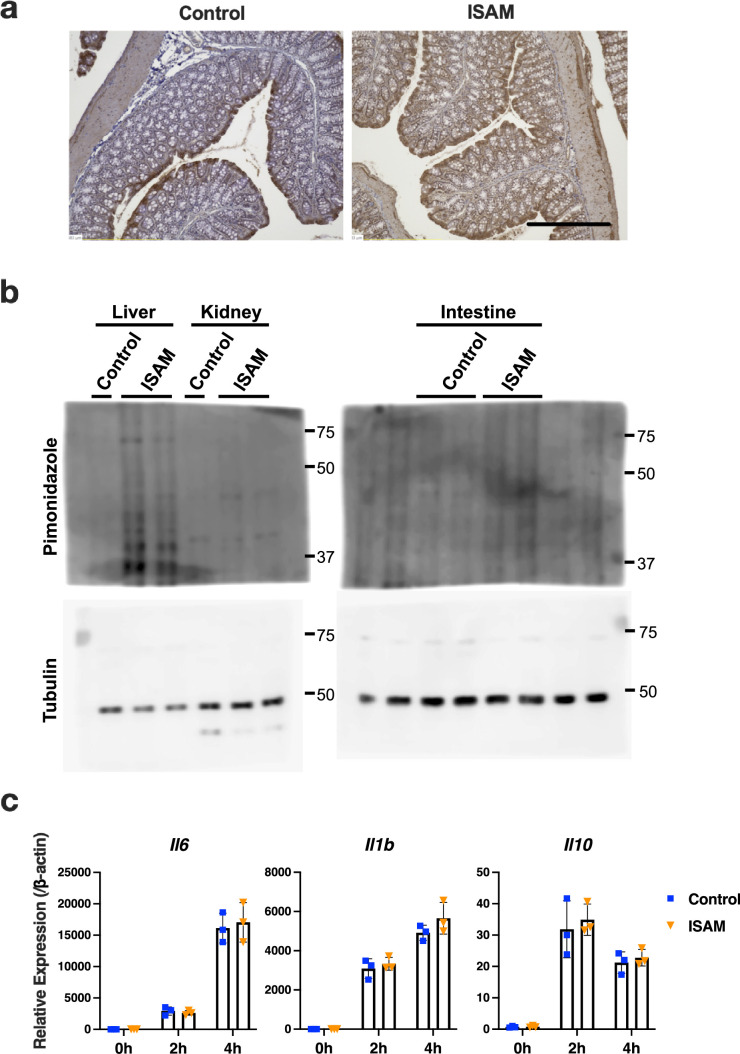
Evaluation of tissue hypoxia in ISAM and expression of cytokine genes in BMDMs after LPS treatment. **a**. Representative pimonidazole staining of intestine from 4 mice for each group. A scale bar corresponds to 483 μm. Each sample was examined twice for technical variability. **b**. Representative pimonidazole immunoblotting of tissue lysates from liver, kidney, and intestine of 4 mice for each group. Each sample was examined for 4 times for technical variability. **c**. BMDMs of ISAM and control mice were differentiated under normoxia. Error bars represent S.E.M. of 3 biological replicates. Source data

To examine the impact of prolonged hypoxia on inflammation, we used a genetically engineered mouse model of systemic hypoxia, inherited super-anaemic mice (ISAM), which exhibit severe anaemia caused by erythropoietin insufficiency and consequent tissue hypoxia (Extended Data Fig. 1a,b)^19,20^. ISAM and control mice were subjected to dextran sulfate sodium (DSS)-induced colitis. ISAM were more susceptible to DSS-induced colitis, showing significant exacerbation of body weight loss (Fig. 1a), colon shortening (Fig. 1b,c) and histopathological damage (Fig. 1d,e). The expression of the proinflammatory cytokine genes, *Il6*, *Il23* and *Il1b*, in colon tissues was higher in ISAM than in control mice with DSS treatment, although *Tnfa* and *Il12b* were similarly increased in the colon tissues of both mouse lines (Fig. 1f). To explore the possibility that these phenotypes in ISAM were attributed to the enhanced proinflammatory response of macrophages, which play a key role in DSS-induced colitis, we collected peritoneal macrophages and examined their response to lipopolysaccharide (LPS) stimulation. The proinflammatory cytokine genes *Il6* and *Il1b* and the anti-inflammatory cytokine gene *Il10* were higher and lower, respectively, in peritoneal macrophages from ISAM than in those from control mice (Fig. 1g). Consistently, peritoneal macrophages of ISAM secreted increased amounts of IL-6 and IL-1β (Fig. 1h). These results suggested that ISAM macrophages were more proinflammatory than control macrophages. To examine whether the proinflammatory phenotypes of ISAM macrophages were acquired due to a hypoxic environment or their intrinsic properties, we cultured bone marrow cells from ISAM and control mice under normoxia and obtained bone-marrow-derived macrophages (BMDMs). The expression levels of *Il6*, *Il1b* and *Il10* in response to LPS were all comparable between BMDMs derived from ISAM and control mice (Extended Data Fig. 1c), suggesting that a prolonged hypoxic environment promoted the proinflammatory phenotypes of ISAM macrophages.

### Macrophages acquire proinflammatory phenotypes in hypoxia

**Fig. 2:**
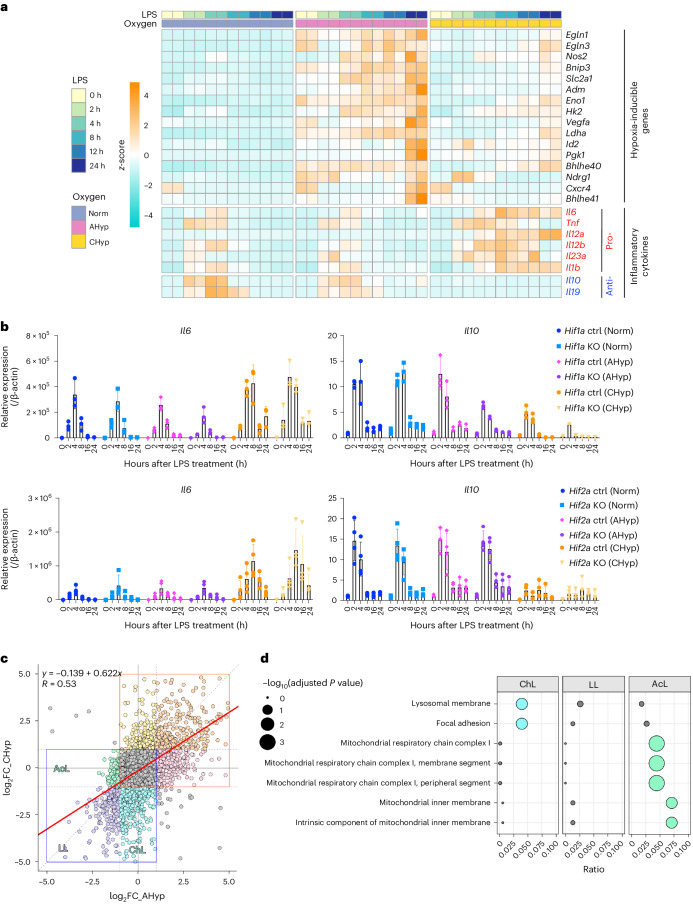
Prolonged hypoxia augments the proinflammatory response of BMDMs. **a**, A heatmap illustrating RNA-seq data of representative HIF target genes and cytokine genes in BMDMs after LPS stimulation. Norm, BMDMs differentiated and stimulated with LPS under normoxia; AHyp, BMDMs differentiated under normoxia and stimulated with LPS under 1% oxygen; CHyp, BMDMs differentiated and stimulated with LPS under 1% oxygen. **b**, Expression of cytokine genes in BMDMs (*n* = 3 biologically independent samples). Norm, AHyp and CHyp BMDMs generated from *Hif1a* mutant mice and *Hif2a* mutant mice were examined. Error bars represent s.e.m. Ctrl, control; KO, knockout. **c**, Scatter-plots showing a correlation of gene expression fold change (FC) by prolonged and acute hypoxia in BMDMs. A horizontal axis indicates log_2_ FC of CHyp versus Norm and a vertical axis indicates log_2_ FC of AHyp versus Norm. Areas enclosed by red and blue squares are those containing upregulated and downregulated genes, respectively. The strength of the correlation was evaluated with a Pearson product-moment correlation coefficient. **d**, Enrichr analysis (Gene Ontology (GO)_Cellular_Component_2017b) of downregulated genes. ChL, LL and AcL indicate gene groups specifically downregulated in CHyp BMDMs, commonly downregulated in CHyp and AHyp BMDMs and specifically downregulated in AHyp BMDMs, respectively, compared with Norm BMDMs after the LPS treatment (indicated in **c**). Circle sizes indicate adjusted *P* values from multiple comparisons and circle colours indicate statistical significance (blue and green, adjusted *P* value < 0.05; grey, not significant). Source data

**Extended Data Fig. 2:**
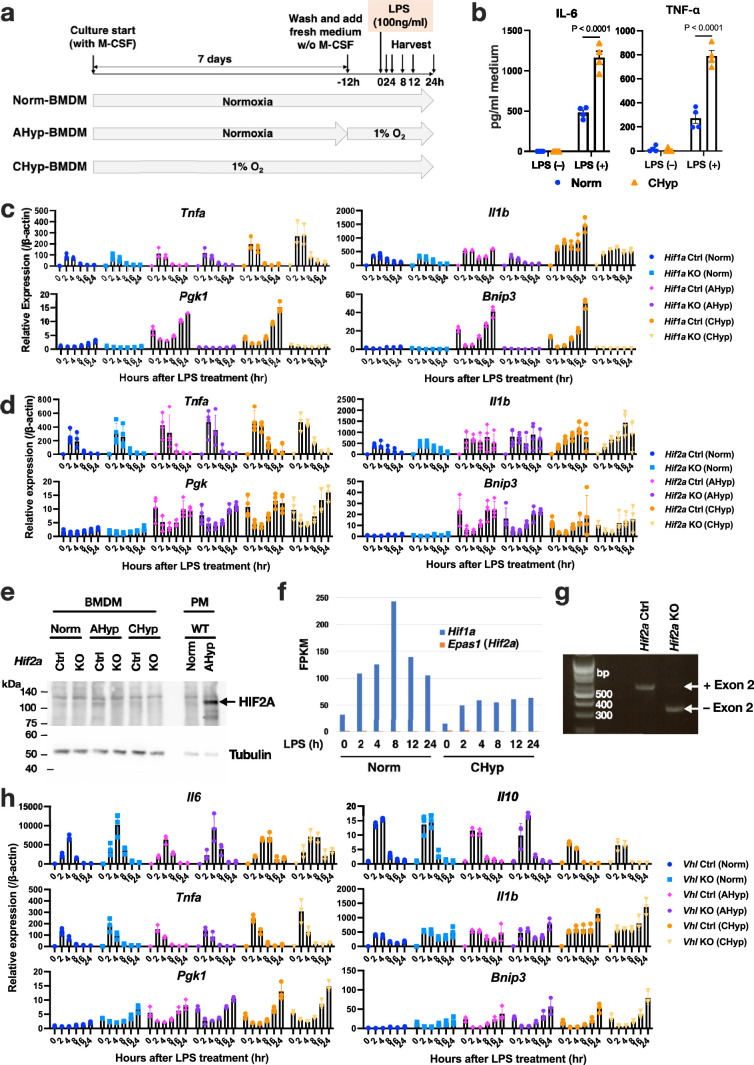
Inflammatory response of BMDMs differentiated under different oxygen tension. **a**. Experimental design for BMDMs differentiation and stimulation with LPS (100 ng/ml). BMDMs were harvested for RNA-seq analysis at 0, 2, 4, 8, 12 and 24 h after LPS treatment. **b**. ELISA of cytokines in culture supernatant of BMDMs stimulated with or without LPS for 12 h (n = 4). Error bars represent S.E.M. Two-way ANOVA was conducted to evaluate statistical significance. **c, d, h**. Gene expression in BMDMs after LPS stimulation (n = 3). Norm, AHyp, and CHyp BMDMs generated from *Hif1a* mutant mice (c), *Hif2a* mutant mice (d), and *Vhl* mutant mice (h) were examined. Error bars represent S.E.M. **e**. Immunoblot analysis of Hif-2α protein. Tubulin was used as a loading control. Peritoneal macrophage (PM) of wild-type mice treated with acute hypoxia (AHyp) was examined as a positive control. A representative result from 2 independent experiments is shown. **f**. FPKM values of *Hif1a* and *Epas1*/*Hif2a* expression obtained from the RNA-seq analysis. **g**. PCR detection of exon 2 deletion of *Hif2a* gene in the genomic DNA of BMDMs after treatment with tamoxifen, performed once. Source data

To verify the impact of oxygen tension on the inflammatory phenotypes of macrophages, we prepared BMDMs differentiated under normoxia and 1% oxygen, which were designated normoxia (Norm) BMDMs and chronic hypoxia (CHyp) BMDMs, respectively (Extended Data Fig. 2a). Norm BMDMs and CHyp BMDMs were subsequently stimulated with LPS under each respective oxygen tension. Acute hypoxia (AHyp) BMDMs were differentiated under normoxia and stimulated with LPS under 1% oxygen (Extended Data Fig. 2a). Inflammatory responses of the three kinds of BMDMs were compared in terms of the LPS-induced transcriptome measured by RNA sequencing (RNA-seq) analysis. Typical HIF target genes were upregulated both in AHyp BMDMs and CHyp BMDMs during LPS stimulation compared with Norm BMDMs (Fig. 2a). Notably, remarkable upregulation and downregulation of proinflammatory and anti-inflammatory genes, respectively, were observed in CHyp BMDMs but not in AHyp BMDMs compared with Norm BMDMs (Fig. 2a). Indeed, IL-6 and tumour necrosis factor-alpha (TNF-α) were more abundantly secreted from CHyp BMDMs than Norm BMDMs in response to LPS (Extended Data Fig. 2b).

We next examined whether the HIF pathway is involved in the gene expression alteration in CHyp BMDMs. Based on a previous study describing that HIF-1α and HIF-2α promote proinflammatory and anti-inflammatory polarization of macrophages, respectively^21^, we examined macrophages deficient in HIF-1α or HIF-2α. *Hif1a* deficiency abolished the expression of *Pgk1* and *Bnip3*, two of the representative HIF target genes, but did not alter proinflammatory gene expression in CHyp BMDMs, except for *Il1b*, which has been reported to be induced by HIF-1α^12^ (Fig. 2b top and Extended Data Fig. 2c). In addition, an anti-inflammatory cytokine gene *Il10* was also downregulated in *Hif1a*-deficient BMDMs (Fig. 2b top). *Hif2a* deficiency did not make any apparent differences in the gene expression in response to LPS (Fig. 2b, bottom, and Extended Data Fig. 2d). To verify the *Hif2a* deletion, we assessed HIF-2α protein levels in BMDMs with wild-type peritoneal macrophages (PMs) treated with acute hypoxia as a positive control, and found that HIF-2α protein abundance was much lower in BMDMs than that in PMs (Extended Data Fig. 2e), which was consistent with our RNA-seq analysis showing much less *Hif2a* expression compared with *Hif1a* (Extended Data Fig. 2f). Nevertheless, HIF-2α protein decrease was apparent in AHyp BMDMs of *Hif2a* knockout mice compared with those of control mice (Extended Data Fig. 2e), and almost complete disruption of the *Hif2a* gene was verified in BMDMs of *Hif2a* knockout mice (Extended Data Fig. 2g). In addition, we compared BMDMs from *Vhl* mutant mice to evaluate an effect of constitutive activation of the HIF pathway, as VHL serves as a substrate recognition subunit of the E3 ubiquitin ligase for HIF proteins. Indeed, *Vhl* deficiency upregulated HIF target genes regardless of oxygen concentration, whereas expression profiles of cytokine genes induced by LPS were similar between control and *Vhl* knockout BMDMs under all conditions, except for *Il1b* upregulation in *Vhl*-deficient Norm BMDMs as *Il1b* is regulated by HIF-1α^12^ (Extended Data Fig. 2h). Thus, we concluded that the proinflammatory phenotypes of BMDMs under prolonged hypoxia were independent of the PHD–HIF pathway.

**Extended Data Fig. 3:**
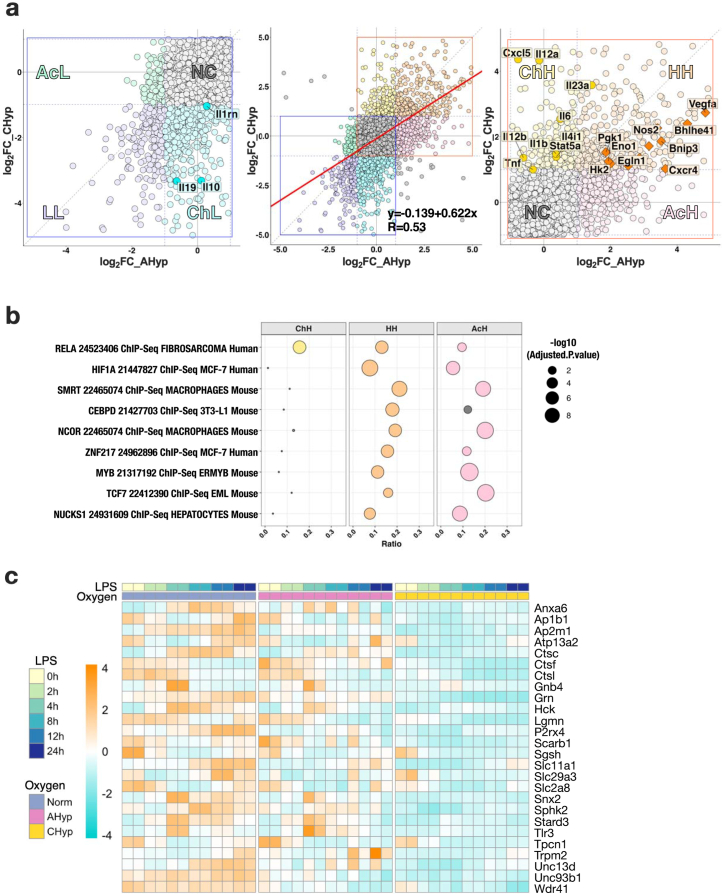
Comparison of LPS-induced transcriptome in BMDMs differentiated under different oxygen tension. **a**. Scatter-plots showing a correlation of gene expression fold changes (AUC ratios) by chronic and acute hypoxia vs. normoxia in BMDMs. A horizontal axis indicates log_2_ fold change of CHyp vs. Norm, and a vertical axis indicates log_2_ fold change of AHyp vs. Norm. Areas enclosed by red and blue squares (middle panel) are highlighted as those containing upregulated and downregulated genes, respectively (right and left panels). ChH: specifically upregulated in CHyp BMDMs, AcH: specifically upregulated in AHyp BMDMs, HH: upregulated in both, ChL: specifically downregulated in CHyp BMDMs, AcL: specifically downregulated in AHyp BMDMs, LL: commonly downregulated in both, NC: not changed. The strength of the correlation was evaluated with Pearson product-moment correlation coefficient. **b**. Enrichr analysis (ChEA 2016) of upregulated genes. ChH, HH and AcH are gene groups indicated in panel A (right). Circle sizes indicate adjusted P values, and circle colours indicate statistical significance (yellow, orange, and pink: adjusted P value < 0.05, grey: not significant). **c**. A heatmap illustrating RNA-seq data of genes that belong to ChL class and assigned to ‘lysosome’ in GO database. Norm: BMDMs differentiated and stimulated with LPS under normoxia, AHyp: BMDMs differentiated under normoxia and stimulated with LPS under 1% O_2_, CHyp: BMDMs differentiated and stimulated with LPS under 1% O_2_.

For a comprehensive understanding of how prolonged and acute hypoxia influence LPS-induced gene expression, we approximated the total amount of transcripts over time as the area under curve (AUC) calculated from messenger RNA level at each time point from 0 to 24 h after LPS addition. AUC ratios of CHyp BMDMs and AHyp BMDMs versus Norm BMDMs were plotted (Fig. 2c and Extended Data Fig. 3a). While overall comparison showed a positive correlation between the impacts of chronic and acute hypoxia on LPS-induced gene expression (*R* = 0.53; Fig. 2c and middle in Extended Data Fig. 3a), genes were categorized into seven classes, specifically upregulated in CHyp BMDMs (ChH), specifically upregulated in AHyp BMDMs (AcH), commonly upregulated in both (HH), specifically downregulated in CHyp BMDMs (ChL), specifically downregulated in AHyp BMDMs (AcL), commonly downregulated in both (LL) and not changed (NC) (Fig. 2c and Extended Data Fig. 3a). As expected from in vivo and ex vivo results of ISAM and control mice, pro- and anti-inflammatory cytokine genes were found in the ChH and ChL classes, respectively (right and left in Extended Data Fig. 3a). Pathway analysis of upregulated genes revealed that genes bound by HIF, SMRT (silencing mediator of retinoic acid and thyroid hormone receptor) and RELA (v-rel avian reticuloendotheliosis viral oncogene homologue A: NF-κB subunit) were enriched in AcH and HH classes, whereas RELA-bound genes were the only significant pathway enriched in the ChH class (Extended Data Fig. 3b). Among downregulated genes, mitochondria-related pathways were enriched in the AcL class (Fig. 2d), which is consistent with a previous report describing that mitochondrial function is dysregulated in acute hypoxia^22^. Notably, the lysosome-related pathway was enriched in the ChL class (Fig. 2d and Extended Data Fig. 3c), implying that lysosomal activity was inhibited in BMDMs exposed to prolonged hypoxia.

### Prolonged hypoxia inhibits lysosomal activity

**Fig. 3:**
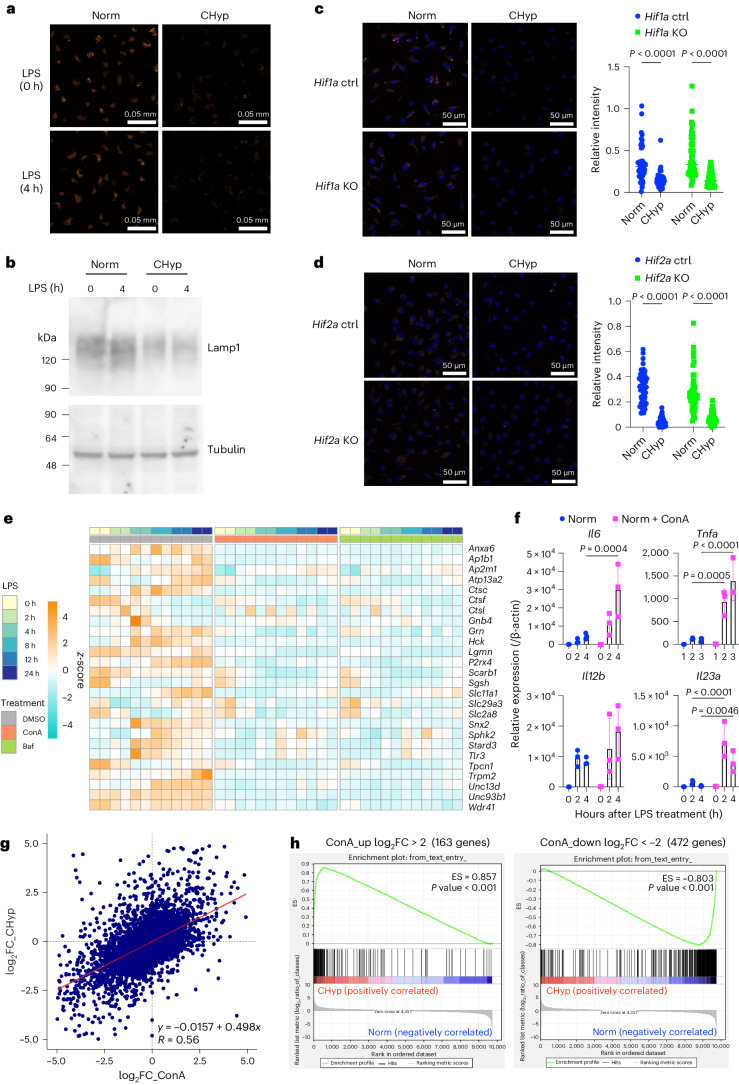
Lysosomal inhibition in prolonged hypoxia and their impacts on the LPS-induced transcriptome. **a**, Representative AcidiFluor ORANGE staining for lysosomal acidification in BMDMs from three independent experiments. **b**, Immunoblot analysis detecting Lamp1 in BMDMs. Tubulin was detected as a loading control. A representative result from three independent experiments is shown. **c**,**d**, Lysosomal acidification in BMDMs generated from *Hif1a* mutant mice (**c**) and *Hif2a* mutant mice (**d**). Representative AcidiFluor ORANGE staining (left) and its quantification (right) from three independent experiments for each. **e**, A heatmap illustrating RNA-seq data of genes that belong to ChL class and assigned to ‘lysosome’ in the GO database, which is the same gene set shown in Extended Data Fig. 3c. Norm BMDMs were treated with concanamycin A (ConA), bafilomycin A1 (Baf) or vehicle (DMSO) at 16 h before LPS stimulation. **f**, Expression of genes upregulated by ConA treatment in BMDMs (*n* = 3 biologically independent samples). Error bars represent s.e.m. **g**, Scatter-plot showing a correlation of gene expression fold changes by prolonged hypoxia and lysosomal inhibition (ConA) in BMDMs. A horizontal axis indicates log_2_ FC of ConA treatment versus DMSO and a vertical axis indicates log_2_ FC of CHyp versus Norm (shown in Fig. 2c). The strength of the correlation was evaluated with a Pearson product-moment correlation coefficient. **h**, GSEA comparing the impacts of prolonged hypoxia with lysosomal inhibition. Gene sets were defined as upregulated (left) or downregulated (right) genes by more than fourfold (log_2_ 4) by ConA treatment. Changes in the LPS-induced transcriptome by prolonged hypoxia were analysed against the gene sets. ES, enrichment score. Scale bars, 50 μm (**a**,**c**,**d**). A two-way ANOVA was conducted to evaluate statistical significance (**c**,**d**,**f**). Source data

**Extended Data Fig. 4:**
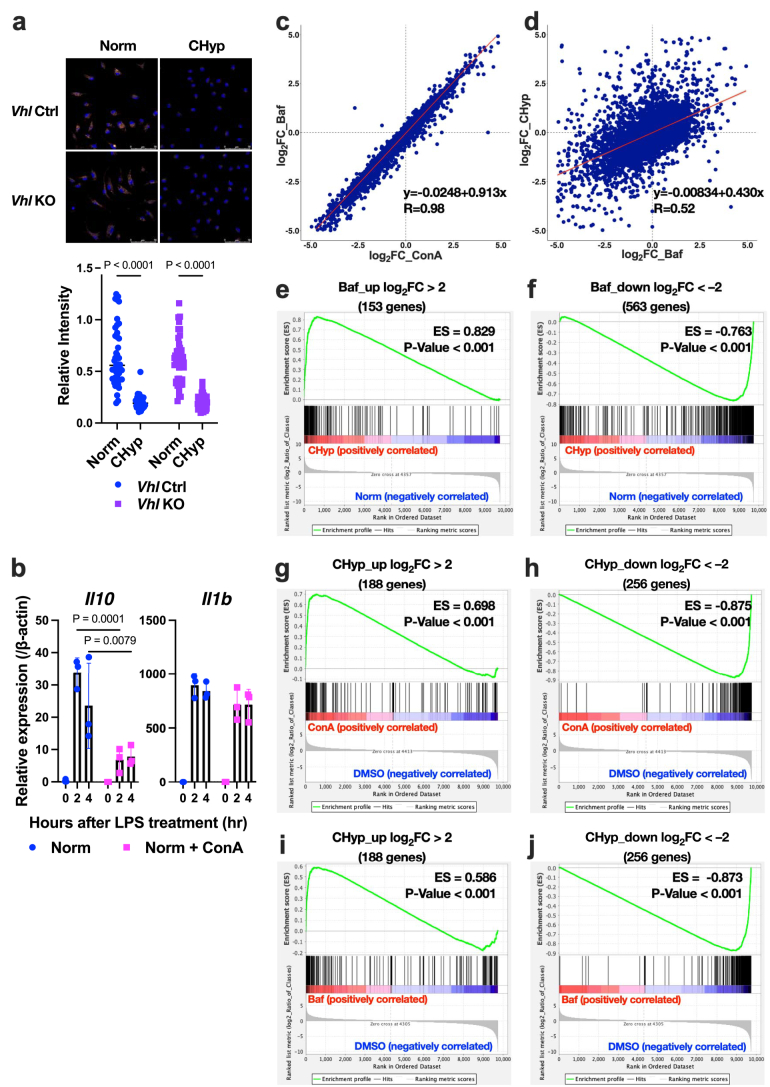
Comparison between impacts of prolonged hypoxia and lysosomal inhibition on the LPS-induced transcriptome. **a**. Lysosomal acidification in BMDMs cultured from *Vhl* mutant mice. Scale bars correspond to 50 μm. Representative AcidiFluor ORANGE staining (top) and its quantification (bottom). Two-way ANOVA was conducted to evaluate statistical significance. A representative result from 3 independent experiments is shown. **b**. Gene expression in BMDMs after LPS stimulation in the presence or absence of ConA (n = 3). Error bars represent S.E.M. Two-way ANOVA was conducted to evaluate statistical significance. **c, d**. Scatter-plots showing correlations of gene expression fold changes (AUC ratios) by ConA and Baf (c) and by prolonged hypoxia and Baf (d) in BMDMs. For panel c, a horizontal axis indicates log_2_ fold change of ConA treatment vs. DMSO, and a vertical axis indicates log_2_ fold change of Baf treatment vs. DMSO. For panel d, a horizontal axis indicates log_2_ fold change of Baf treatment vs. DMSO, and a vertical axis indicates log_2_ fold change of CHyp vs. Norm (shown in Fig. 2c). The strength of each correlation was evaluated with Pearson product-moment correlation coefficient. Norm: BMDMs differentiated and stimulated with LPS under normoxia, CHyp: BMDMs differentiated and stimulated with LPS under 1% O_2_. **e, f**. Gene set enrichment analysis comparing the impacts of prolonged hypoxia with lysosomal inhibition (Baf). Gene sets were defined as upregulated (e) or downregulated (f) genes by more than 4-fold (log_2_ 4) by Baf treatment. Changes in the LPS-induced transcriptome by prolonged hypoxia were analysed against the gene sets. **g-j**. Gene set enrichment analysis comparing the impacts of lysosomal inhibition with prolonged hypoxia. Gene sets were defined as upregulated (g, i) or downregulated (h, j) genes by more than 4-fold (log_2_ 4) by prolonged hypoxia. Changes in the LPS-induced transcriptome by lysosomal inhibitors, ConA (g, h) and Baf (i, j), were analysed against the gene sets. Source data

Because decline of lysosomal activity seemed to accompany the proinflammatory phenotypes of BMDMs under prolonged hypoxia, we examined lysosomal acidification in Norm BMDMs and CHyp BMDMs. Fluorescence intensity indicating the lysosomal acidification was decreased in CHyp BMDMs compared with Norm BMDMs irrespective of LPS treatment (Fig. 3a). Consistently, CHyp BMDMs exhibited reduction in the protein level of Lamp1, which is a lysosomal membrane protein (Fig. 3b). These results suggest that prolonged hypoxia inhibits lysosomal activity. *Hif1a*, *Hif2a* and *Vhl* deficiency did not affect the lysosomal inhibition by prolonged hypoxia, indicating that HIF activity is dispensable for and does not interfere with the lysosomal response to prolonged hypoxia (Fig. 3c,d and Extended Data Fig. 4a).

We questioned to what extent lysosomal inhibition contributed to the transcriptome alteration under prolonged hypoxia. To compare the impacts of lysosomal inhibition on the LPS-induced transcriptome with those of prolonged hypoxia, we conducted RNA-seq analysis of BMDMs differentiated under normoxia and treated with lysosomal inhibitors, concanamycin A (ConA) and bafilomycin A1 (Baf) or vehicle (dimethylsulfoxide (DMSO)) and examined how the lysosomal inhibitors influence the LPS-induced transcriptome. The lysosome-related pathway genes that were identified in the ChL class (Extended Data Fig. 3c) were confirmed to decrease in both ConA-treated and Baf-treated BMDMs (Fig. 3e). Proinflammatory cytokine genes in the ChH class, except for *Il1b* and *Il10* in the ChL class were upregulated and downregulated by ConA treatment, respectively (Fig. 3f and Extended Data Fig. 4b).

We calculated AUC ratios of gene expression in ConA-treated and Baf-treated BMDMs versus DMSO-treated BMDMs and verified that changes in the gene expression profiles induced by the two lysosomal inhibitors were highly matched (Extended Data Fig. 4c). The AUC ratios of ConA-treated and Baf-treated versus DMSO-treated BMDMs were positively correlated with those of CHyp BMDMs versus Norm BMDMs (Fig. 3g and Extended Data Fig. 4d). To further examine the similarity between them, Gene Set Enrichment Analysis (GSEA) was performed. Gene sets defined as upregulated and downregulated genes by ConA treatment were strongly enriched in the genes upregulated and downregulated by prolonged hypoxia, respectively (Fig. 3h). Similar results were obtained with gene sets defined by Baf treatment (Extended Data Fig. 4e,f). Conversely, gene sets defined as upregulated and downregulated genes by prolonged hypoxia were strongly enriched in the genes upregulated and downregulated by treatment with the lysosomal inhibitors, respectively (Extended Data Fig. 4g–j). These results indicated a remarkable similarity between the effects of prolonged hypoxia and lysosomal inhibition on the LPS-induced transcriptional responses of macrophages, which let us suppose that lysosomal inhibition caused by prolonged hypoxia is responsible for proinflammatory phenotypes of macrophages.

**Extended Data Fig. 5:**
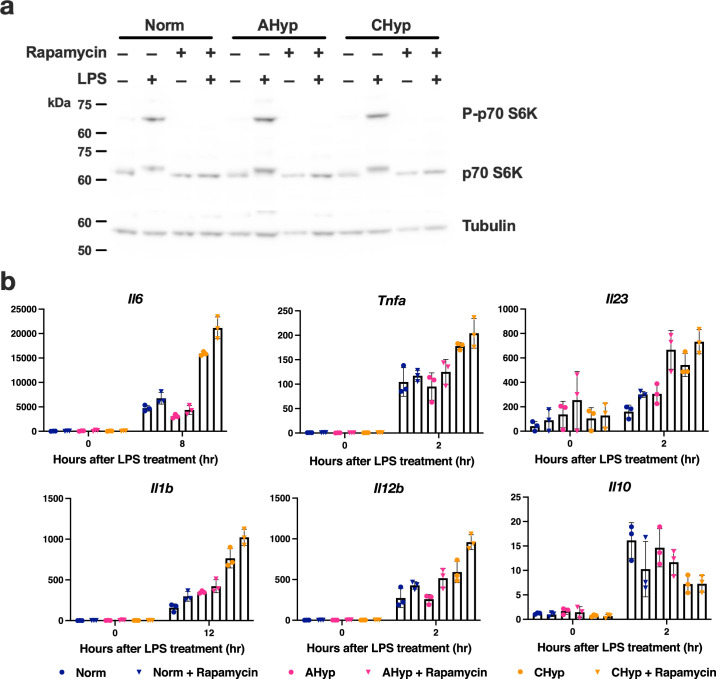
Involvement of mTOR pathway in inflammatory response of BMDM under different oxygen tension. **a**. Phosphorylation status of p70 S6K was examined to evaluate mTOR pathway activity. LPS treatment increased p70 S6K phosphorylation (P-p70 S6K), which was decreased by mTOR inhibitor rapamycin regardless of the oxygen tension. Norm: BMDMs differentiated and stimulated with LPS under normoxia, AHyp: BMDMs differentiated under normoxia and stimulated with LPS under 1% O_2_, CHyp: BMDMs differentiated and stimulated with LPS under 1% O_2_. A representative result from 2 independent experiments is shown. **b**. Gene expression in BMDMs after LPS stimulation (n = 3). Norm, AHyp, and CHyp BMDMs treated with or without rapamycin were examined. Error bars represent S.E.M. of 3 biological replicates. Source data

A previous report suggested that short-term hypoxia (6 h) impairs lysosomal activity through the suppression of mTOR pathway in human umbilical vein endothelial cells (HUVECs)^23^; however, in LPS-treated BMDMs, mTOR activity estimated by p70 S6K phosphorylation remained unaffected by both chronic and acute hypoxia (Extended Data Fig. 5a). Furthermore, mTOR inhibition by rapamycin in Norm BMDMs did not induce cytokine gene upregulation as observed in CHyp BMDMs (Extended Data Fig. 5b). These results suggest that chronic hypoxia inhibits lysosomal activity and alters cytokine gene expression independently of mTOR activity in BMDMs.

### Prolonged hypoxia suppresses vitamin B6 bioactivation

**Extended Data Fig. 6:**
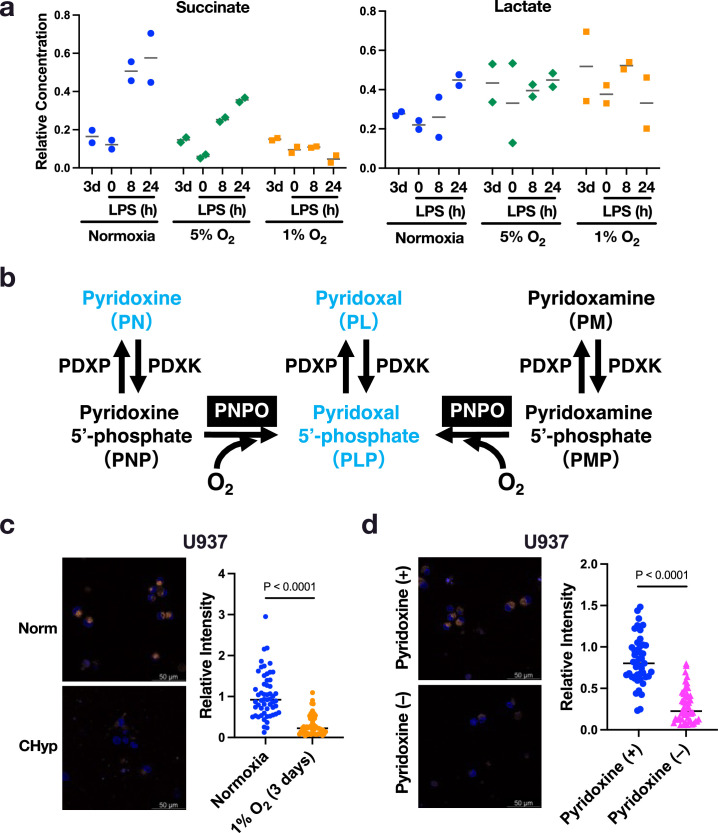
Metabolite changes induced by prolonged hypoxia and its effect on lysosomal activity. **a**. Relative amounts of cellular succinate and lactate in the BMDMs differentiated and stimulated with LPS under normoxia, 5% O_2_ and 1% O_2_ (n = 2). The metabolites were also measured on day 3 of differentiation. **b**. Schematic illustration indicating vitamin B6 metabolism and catalysing enzymes. PDXP: pyridoxal phosphatase, PDXK: pyridoxal kinase, PNPO: pyridoxine 5’-phosphate oxidase. **c, d**. Lysosomal acidification in PMA-treated U937 cells under different oxygen tension (c) and with or without pyridoxine in the culture medium (d). Scale bars correspond to 50 μm. Representative AcidiFluor ORANGE staining (left) and its quantification (right). Two-sided Student’s *t*-test was conducted to evaluate statistical significance. A representative result from 3 independent experiments is shown for each. Source data

To explore the mechanism by which prolonged hypoxia suppresses lysosomal activity in BMDMs, we examined metabolome of Norm BMDMs and CHyp BMDMs together with BMDMs differentiated under 5% oxygen. We expected that increased levels of succinate and lactate in CHyp BMDMs could give us a clue to lysosomal dysfunction because succinate has been reported as a metabolite inducing the proinflammatory status of macrophages^10,24^ and because hypoxia-induced lactate accumulation mediates macrophage polarization through histone modification^25^; however, neither succinate nor lactate increased in CHyp BMDMs, and rather, the abundance of succinate after LPS treatment incrementally decreased according to the oxygen tension (Extended Data Fig. 6a).

**Fig. 4:**
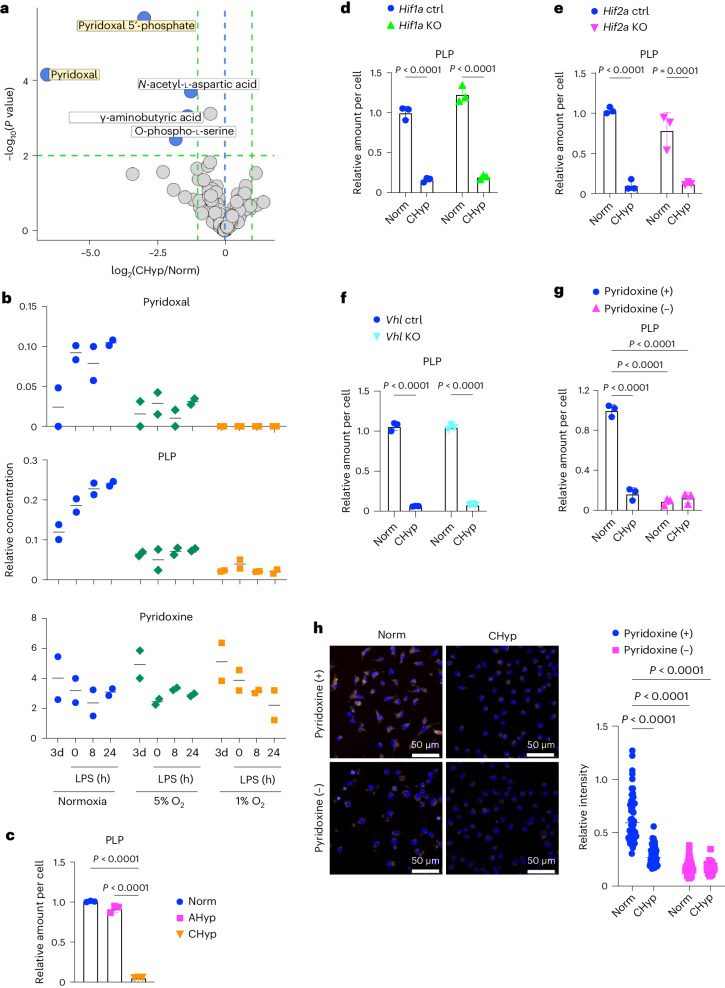
Prolonged hypoxia reduces PLP. **a**, Volcano plot showing metabolome comparison between CHyp BMDMs and Norm BMDMs. A horizontal dashed line indicates a significance threshold (*P* = 0.01) and green vertical dashed lines indicate levels of twofold increase and decrease. The blue vertical dashed line indicates the level at which CHyp and Norm are equal. A two-sided Welch’s *t*-test was conducted for calculating *P* values. **b**, Relative amounts of pyridoxal, PLP and pyridoxine in BMDMs differentiated and stimulated with LPS under normoxia, 5% and 1% oxygen (*n* = 2 biologically independent samples). The metabolites were also measured on day 3 of differentiation. **c**–**g**, Relative amount of PLP in Norm BMDM, AHyp BMDM and CHyp BMDM (**c**) and Norm BMDM and CHyp BMDM generated from *Hif1a* mutant mice (**d**), *Hif2a* mutant mice (**e**), *Vhl* mutant mice (**f**) and Norm BMDM and CHyp BMDM with or without pyridoxine in the culture medium (**g**) (*n* = 3 biologically independent samples for each experiment). Error bars represent s.e.m. **h**, Lysosomal acidification in Norm BMDMs and CHyp BMDMs with or without pyridoxine in the culture medium. Scale bars, 50 μm. Representative AcidiFluor ORANGE staining (left) and its quantification (right) from three independent experiments. A two-way ANOVA (**d**–**h**) and one-way ANOVA (**c**) were conducted to evaluate statistical significance. Source data

As a comprehensive evaluation of how prolonged hypoxia influences the LPS-induced changes in cellular metabolites, a volcano plot was drawn to determine differential metabolites in CHyp BMDMs versus Norm BMDMs during LPS treatment (Fig. 4a). Out of five metabolites of statistical significance, pyridoxal and PLP were dramatically decreased in CHyp BMDMs (Fig. 4a) and incrementally decreased according to the oxygen tension (Fig. 4b). In good contrast, pyridoxine, which is contained in the culture medium as the main source of pyridoxal and PLP (Extended Data Fig. 6b), showed no changes among all oxygen conditions (Fig. 4b). Cellular PLP levels were dramatically reduced by chronic hypoxia but not by acute hypoxia in BMDMs (Fig. 4c). A recent report described that HIF-1 upregulates PDXP (Extended Data Fig. 6b), resulting in the enhanced degradation of PLP^26^. To evaluate the HIF pathway involvement in the PLP decrease in prolonged hypoxia, we measured cellular PLP in *Hif1a*-, *Hif2a*- and *Vhl-*deficient BMDMs. Prolonged hypoxia decreased PLP irrespective of the HIF activity (Fig. 4d–f), indicating that PLP decrease induced by prolonged hypoxia is independent of the HIF activity.

To examine whether PLP is required for the maintenance of lysosomal activity, we cultured BMDMs in the medium without pyridoxine and examined lysosomal acidification. Pyridoxine restriction reduced the cellular PLP and inhibited the lysosomal acidification in Norm BMDMs to the extent comparable to CHyp BMDMs (Fig. 4g,h). Similarly, pyridoxine restriction inhibited the lysosomal acidification in phorbol 12-myristate 13-acetate (PMA)-treated U937 cells as observed in those exposed to chronic hypoxia (Extended Data Fig. 6c,d).

**Extended Data Fig. 7:**
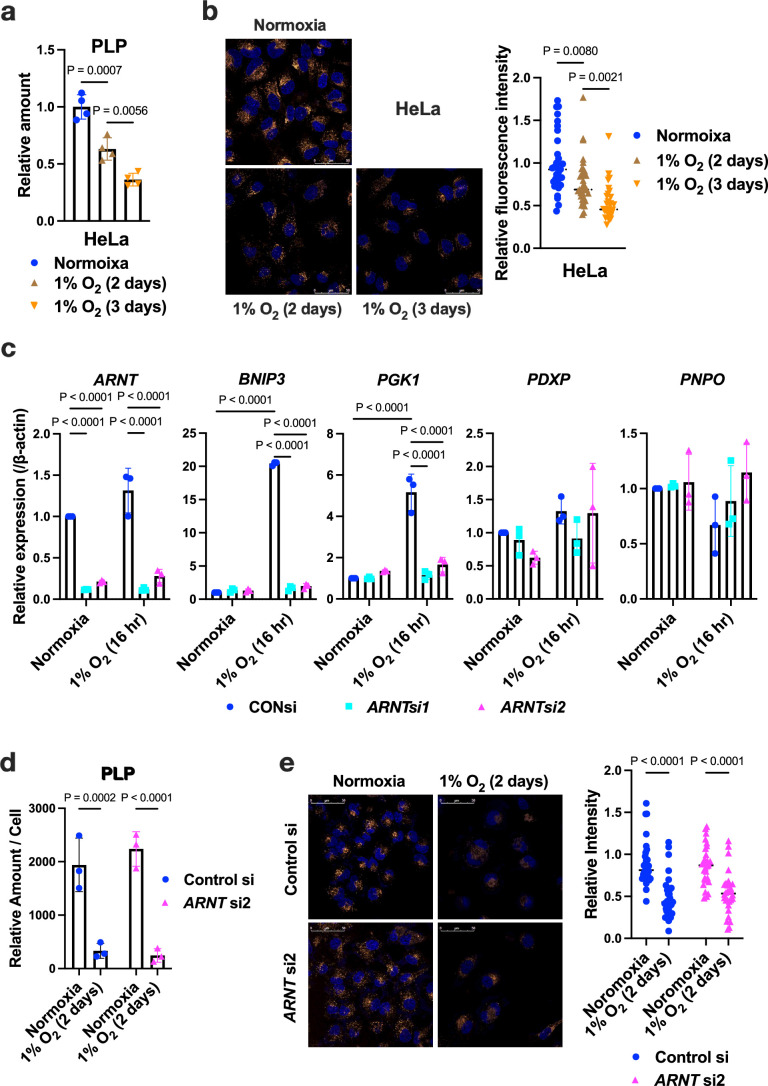
Impact of prolonged hypoxia on PLP levels and lysosomal acidification in HeLa cells. **a**. PLP levels in HeLa cells in normoxia and hypoxia for 2 and 3 days (n = 4 biologically independent samples). **b**. Lysosomal acidification in HeLa cells. Representative AcidiFluor ORANGE staining (left) and its quantification (right). A representative result from 3 independent experiments is shown. **c**. Gene expression in HeLa cells with or without *ARNT* knockdown in normoxia and hypoxia for 16 hours (n = 3). **d**. PLP levels in HeLa cells with or without *ARNT* knockdown in normoxia and hypoxia for 2 days (n = 3). **e**. Lysosomal acidification in HeLa cells with or without *ARNT* knockdown. Representative AcidiFluor ORANGE staining (left) and its quantification (right) from 3 independent experiments. Scale bars correspond to 50 μm (b, e). Error bars represent S.E.M. (a, c, d). One-way ANOVA (a, b) and two-way ANOVA (c-e) were conducted to evaluate statistical significance. Source data

To investigate an impact of prolonged hypoxia on PLP levels and lysosomal activity in cell types other than BMDMs, we measured PLP levels in HeLa cells during hypoxia. The inhibition of lysosomal acidification progressed along with the decrease in PLP during 3 days of hypoxic exposure (Extended Data Fig. 7a,b). To examine the HIF pathway independency, we knocked down the *ARNT* gene, which encodes an essential heterodimeric partner molecule of HIF-1α and HIF-2α, in HeLa cells and verified the lack of induced expression of HIF target genes, *BNIP3* and *PGK1*, in response to hypoxia (Extended Data Fig. 7c). Consistent with observations in BMDMs, no discernible effects on PLP reduction and lysosomal activity were observed in *ARNT* knockdown cells under prolonged hypoxia (Extended Data Fig. 7d,e).

### PNPO generates PLP in response to oxygen

**Fig. 5:**
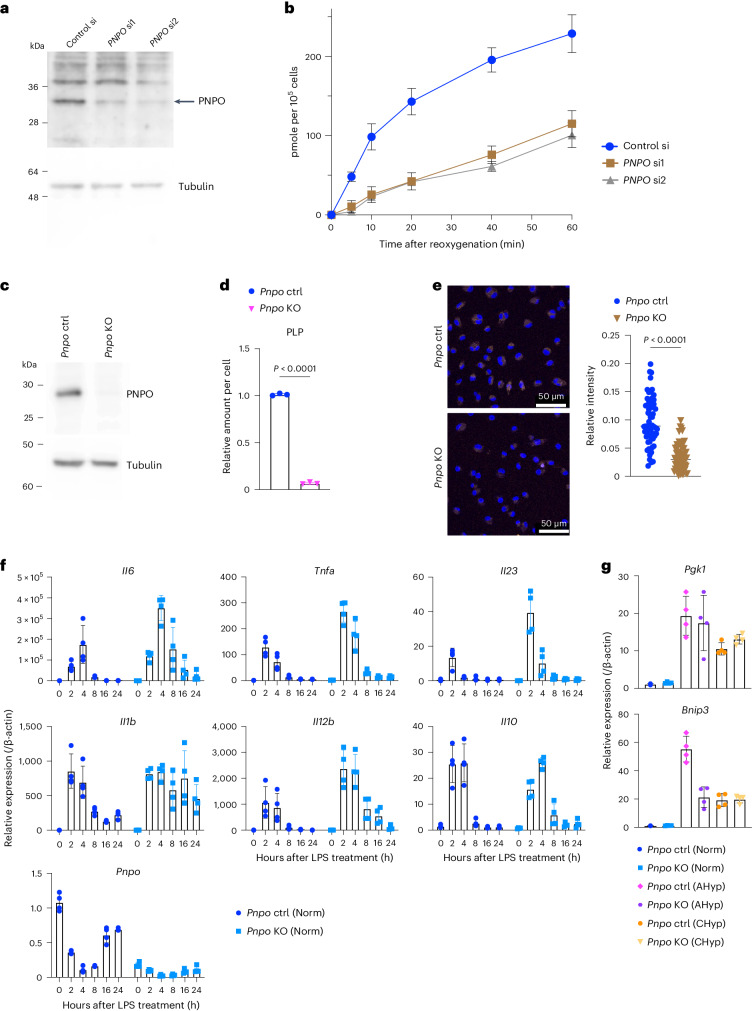
PNPO generates PLP in response to oxygen. **a**, Immunoblot analysis detecting PNPO in U937 cells treated with PNPO siRNAs or control siRNA. Tubulin was detected as a loading control. A representative result from three independent experiments is shown. **b**, PLP increase in U937 cells pre-exposed to 1% oxygen. PLP quantity upon reoxygenation is plotted (*n* = 3 biologically independent samples for each experiment). **c**, Immunoblot analysis detecting PNPO protein in BMDM generated from *Pnpo* mutant mice. A representative result from two independent experiments is shown. **d**, PLP levels in *Pnpo*-deficient Norm BMDMs (*n* = 3 biologically independent samples). **e**, Lysosomal acidification in *Pnpo*-deficient Norm BMDMs. Scale bars, 50 μm. Representative AcidiFluor ORANGE staining (left) and its quantification (right) from three independent experiments. **f**,**g**, Gene expression in response to LPS in normoxia (**f**) and in response to acute and chronic hypoxia without LPS (**g**) in *Pnpo*-deficient BMDMs (*n* = 4 biologically independent samples). Error bars represent s.e.m. (**b**,**d**,**f**,**g**). A two-sided Student’s *t*-test (**d**,**e**) was conducted to evaluate statistical significance. Source data

To address the molecular mechanism regulating PLP bioavailability in response to prolonged hypoxia, we focused on the synthesis of PLP from pyridoxine, which is catalysed by PNPO (Extended Data Fig. 6b). Because PNPO requires oxygen as a substrate, continuous hypoxic condition was expected to block the reaction catalysed by PNPO resulting in the depletion of PLP and pyridoxal. We examined PNPO contribution to the oxygen-dependent synthesis of PLP using 1% oxygen pre-exposed U937 cells with or without *PNPO* knockdown (Fig. 5a). The PLP increase upon reoxygenation was blunted in *PNPO*-knockdown cells (Fig. 5b), indicating that PNPO serves as an oxygen sensor regulating PLP bioavailability.

To evaluate the requirement of the PNPO–PLP axis for the response to chronic hypoxia in macrophages, we cultured BMDMs from *Pnpo* mutant mice and verified complete loss of the PNPO protein (Fig. 5c). The *Pnpo*-deficient BMDMs exhibited a reduction in PLP, inhibition of lysosomal acidification and enhanced expression of proinflammatory cytokine genes (Fig. 5d–f). Expression of HIF target genes was not influenced by *Pnpo* deficiency in CHyp BMDMs (Fig. 5g). These results suggest that the PNPO–PLP axis is a key to shaping inflammatory phenotypes in macrophages under chronic hypoxia.

### Lysosomal inhibition in hypoxia reduces Fe^2+^ and TET2

**Fig. 6:**
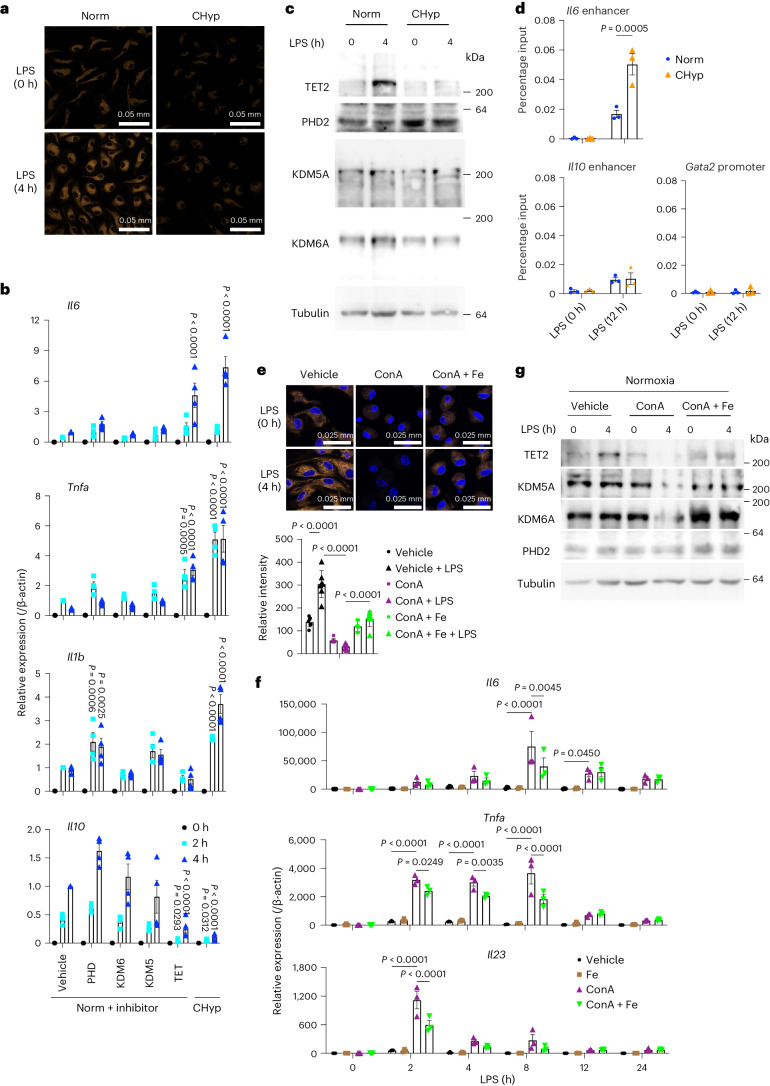
Prolonged hypoxia reduces Fe^2+^ availability and abrogates LPS-induced TET2 protein accumulation. **a**, Representative FerroOrange staining for intracellular Fe^2+^ in BMDMs from three independent experiments. **b**, Expression of cytokine genes in BMDMs after LPS stimulation (*n* = 4 biologically independent samples). Norm + inhibitor, BMDMs differentiated under normoxia in the presence of 2OG-dependent dioxygenase inhibitors or vehicle (DMSO) and stimulated with LPS under normoxia. **c**, Immunoblot analysis detecting 2OG-dependent dioxygenases in BMDMs. Tubulin was detected as a loading control. A representative result from three independent experiments is shown. **d**, H3K27ac deposition detected by ChIP assay (*n* = 3 biologically independent samples). The *Gata2* promoter region was evaluated as a negative control locus. **e**–**g**, Norm BMDMs treated with ConA or vehicle with or without ferric ammonium citrate (Fe) were examined by FerroOrange staining for intracellular Fe^2+^ (*n* = 6 biologically independent samples) (**e**), cytokine gene expression (*n* = 3 biologically independent samples) (**f**), and immunoblot analysis detecting 2OG-dependent dioxygenases as a representative result from two independent experiments is shown (**g**). Scale bars, 50 μm (**a**) and 25 μm (**e**). Error bars represent s.e.m. (**b**,**d**,**f**) and s.d. of seven fields per sample (**e**). A two-way ANOVA was conducted to evaluate statistical significance (**b**,**d**–**f**). Comparisons were made against Norm + vehicle (**b**). Source data

We next investigated a molecular mechanism linking the lysosomal inhibition and proinflammatory phenotypes of macrophages under prolonged hypoxia. Because a recent study demonstrated that lysosomes are organelles that regulate cellular ferrous iron (Fe^2+^) levels and that their dysfunction causes cellular Fe^2+^ deficiency^27^, we expected that prolonged hypoxia reduced Fe^2+^ availability due to the lysosomal inhibition. Indeed, an intracellular level of Fe^2+^ detected by a fluorescent probe was remarkably decreased in CHyp BMDMs irrespective of LPS treatment (Fig. 6a).

**Extended Data Fig. 8:**
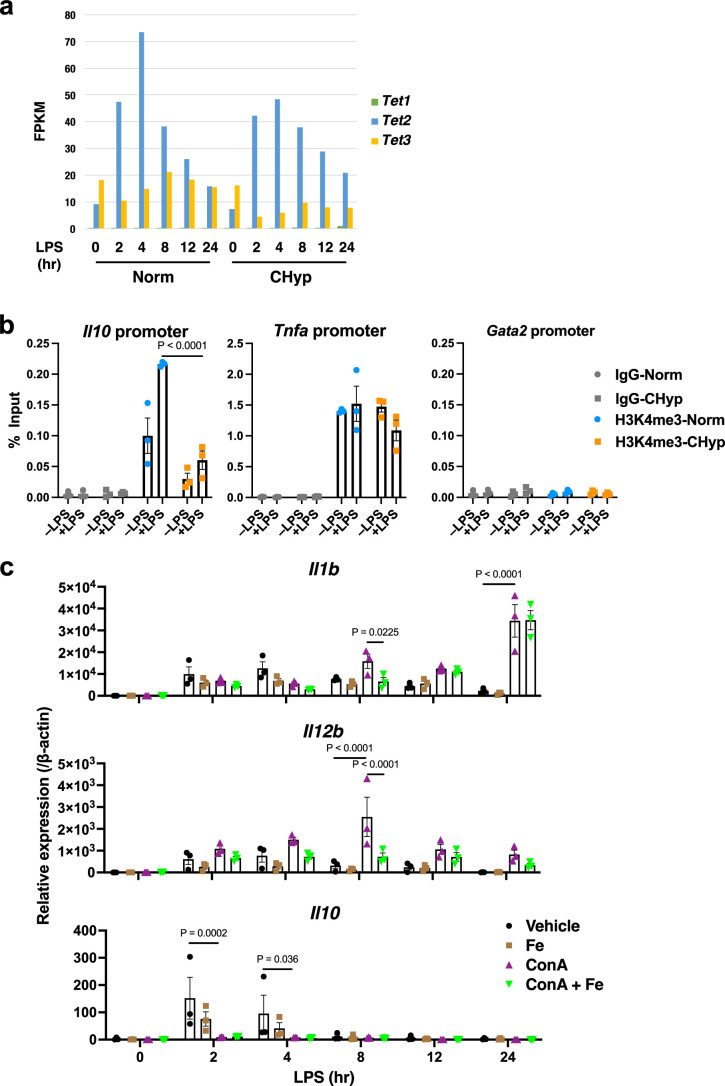
LPS-induced gene expression and H3K4 trimethylation in BMDM. **a**. FPKM values of *Tet1*, *Tet2* and *Tet3* expression obtained from the RNA-seq analysis. Norm: BMDMs differentiated and stimulated with LPS under normoxia, CHyp: BMDMs differentiated and stimulated with LPS under 1% O_2_. **b**. H3K4me3 deposition at the *Il10* promoter and *Tnfa* promoter in BMDMs treated with or without LPS for 12 h (n = 3). The *Gata2* promoter was evaluated as a negative control locus. **c**. Expression of cytokine genes in BMDMs after LPS stimulation (n = 3). BMDMs differentiated under normoxia were treated with 10 nM ConA or vehicle (DMSO) with or without 0.1 mg/ml ferric ammonium citrate at 16 h before LPS stimulation. Error bars represent S.E.M. (b, c). Two-way ANOVA was conducted to evaluate statistical significance (b, c). Source data

Among proteins that require Fe^2+^ for their activities, we focused on 2OG-dependent dioxygenases because they were reported to serve as oxygen sensors by switching off their activities under hypoxic conditions^3,8,9^, expecting that not only oxygen but also Fe^2+^ availability could regulate their enzymatic activities and that decline of their enzymatic activities due to Fe^2+^ limitation was responsible for the proinflammatory phenotypes of CHyp BMDMs. To test this possibility, BMDMs were differentiated under normoxia in the presence of inhibitors against 2OG-dependent dioxygenases, PHD, KDM6A, KDM5A and TET, and stimulated with LPS (Fig. 6b). We found that the TET inhibitor mimicked the effects of prolonged hypoxia on the gene expression of *Il6*, *Tnfa* and *Il10*. Hyperactivation of *Il1b* in CHyp BMDMs was mimicked by PHD inhibition but not by TET inhibition, which is consistent with a previous report that HIF-1α induces *Il1b* expression^12^. As TET2 turned out to be an isoform that was mainly expressed in BMDMs and transcriptionally induced by LPS treatment in both Norm BMDMs and CHyp BMDMs (Extended Data Fig. 8a), we focused on TET2 for analysis of TET protein function hereafter.

We checked the protein amount of TET2 together with PHD2, KDM5A and KDM6A in BMDMs with or without LPS treatment (Fig. 6c). TET2 protein robustly accumulated in response to LPS in Norm BMDMs in accordance with the increase in its mRNA, but to our surprise, the protein accumulation was almost abrogated in CHyp BMDMs despite the LPS-induced mRNA increase (Fig. 6c and Extended Data Fig. 8a). Because TET proteins possess a high *K*_m_ value for Fe^2+^ compared with other 2OG-dependent dioxygenases^10^, TET proteins may be destabilized by losing Fe^2+^ under the prolonged hypoxia where Fe^2+^ availability is limited.

TET2 inactivation has been considered to underlie the proinflammatory milieu, as *Tet2* knockout mice are susceptible to DSS-induced colitis with hyperactivation of *Il6* during inflammation^28^. Recognizing that TET2 mediates DNA demethylation, we compared the LPS-induced methylome between Norm BMDMs and CHyp BMDMs but could not find any significant differences (data not shown). Another role of TET2 has been reported to recruit HDAC for limiting deposition of acetylated histones at the *Il6* locus in response to LPS^28^. Consistently, CHyp BMDMs mimicked *Tet2*-deficient macrophages, showing increased deposition of acetylated histone H3K27 (H3K27ac) in the *Il6* enhancer region after LPS treatment (Fig. 6d). Although *Il10* expression was suppressed in CHyp BMDMs, H3K27ac deposition at the *Il10* enhancer region was not changed (Fig. 6d). Instead, we found that trimethylation of H3K4 (H3K4me3) was decreased at the *Il10* promoter in CHyp BMDMs (Extended Data Fig. 8b). Because the TET inhibitor downregulated *Il10* in Norm BMDMs (Fig. 6b), we speculate that loss of TET2 accumulation is one of the causes for the *Il10* downregulation in CHyp BMDMs, resulting in the epigenetic regulation at the *Il10* promoter.

Consistent with a previous report^27^, lysosomal inhibition by ConA treatment reduced cellular Fe^2+^ levels in BMDMs under normoxia (Fig. 6e). The ConA treatment also remarkably increased and decreased proinflammatory and anti-inflammatory gene expression in response to LPS, respectively (Fig. 6f and Extended Data Fig. 8c), accompanied by loss of TET2 protein accumulation (Fig. 6g). Exogenous iron supplementation partially recovered cellular Fe^2+^ in BMDMs treated with ConA (Fig. 6e). Consistent with the partial recovery of Fe^2+^, the gene expression and TET2 protein levels affected by the ConA treatment were reversed but partially (Fig. 6f,g and Extended Data Fig. 8c). These results suggest that lysosomal inhibition caused by prolonged hypoxia reduces Fe^2+^ availability, resulting in the abrogation of LPS-induced TET2 protein accumulation and enhancement of proinflammatory gene expression.

### Pyridoxal reverses phenotypes of prolonged hypoxia

**Fig. 7:**
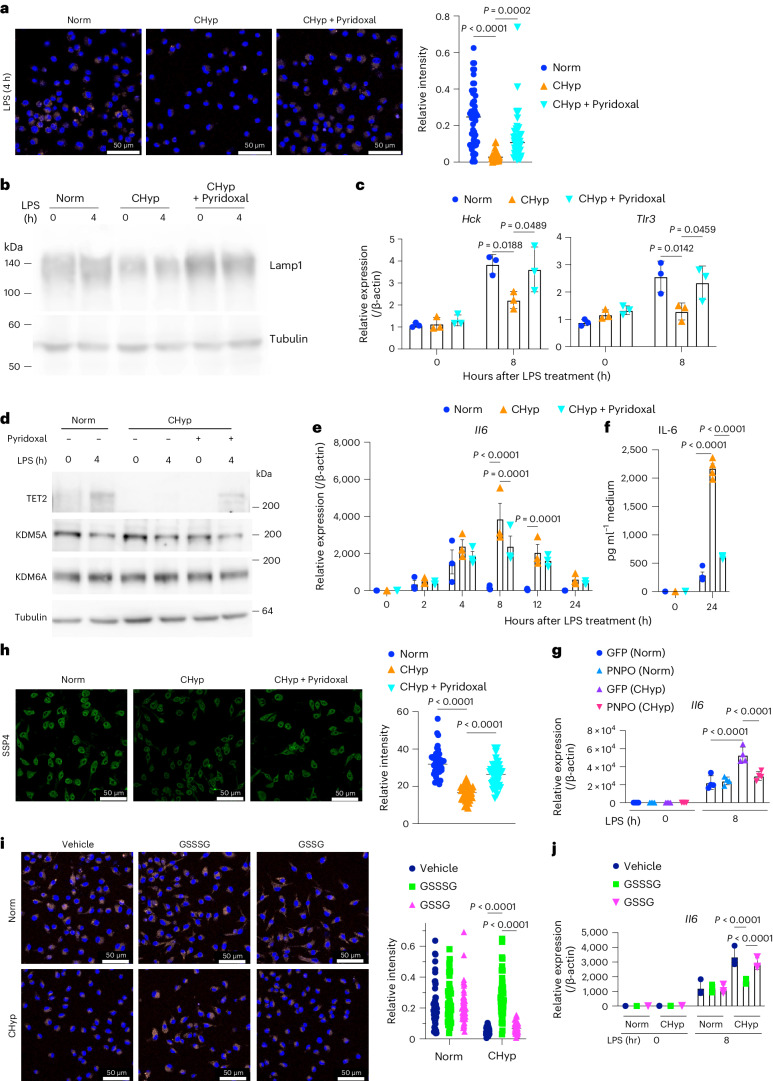
Pyridoxal and supersulfides reverse enhanced inflammatory response of macrophages in prolonged hypoxia. **a**, Lysosomal acidification in BMDMs. Representative AcidiFluor ORANGE staining (left) and its quantification (right) from three independent experiments. CHyp + Pyridoxal, BMDMs differentiated under 1% oxygen in the presence of 50 μg ml^−1^ pyridoxal. **b**. Immunoblot analysis detecting Lamp1 in BMDMs. Tubulin was detected as a loading control. A representative result from three independent experiments is shown. **c**, Expression of representative lysosome-related genes in BMDMs (*n* = 3 biologically independent samples). **d**, Immunoblot analysis detecting 2OG-dependent dioxygenases in BMDMs. Tubulin was detected as a loading control. A representative result from three independent experiments is shown. **e**, Relative *Il6* expression in BMDMs (*n* = 3 biologically independent samples). **f**, ELISA of IL-6 in the culture supernatant of BMDMs (*n* = 4 biologically independent samples). **g**, *Il6* expression in BMDM with or without PNPO overexpression (*n* = 4 biologically independent samples). **h**, SSP4 staining to detect supersulfides in BMDM. Representative SSP4 staining (left) and quantification (right) from three independent experiments. **i**, Effects of GSSSG, a supersulfide donor, and GSSG on lysosomal acidification. Representative AcidiFluor ORANGE staining (left) and its quantification (right) from three independent experiments. Oxidized glutathione, GSSG, was added as a negative control. **j**, *Il6* expression in BMDM treated with GSSSG or GSSG (*n* = 3 biologically independent samples). Scale bars, 50 μm (**a**,**h**,**i**). Error bars represent s.e.m. (**c**,**e**–**g**,**j**). One-way ANOVA (**a**,**h**) and two-way ANOVA (**c**,**e**–**g**,**i**,**j**) were conducted to evaluate statistical significance. Source data

**Extended Data Fig. 9:**
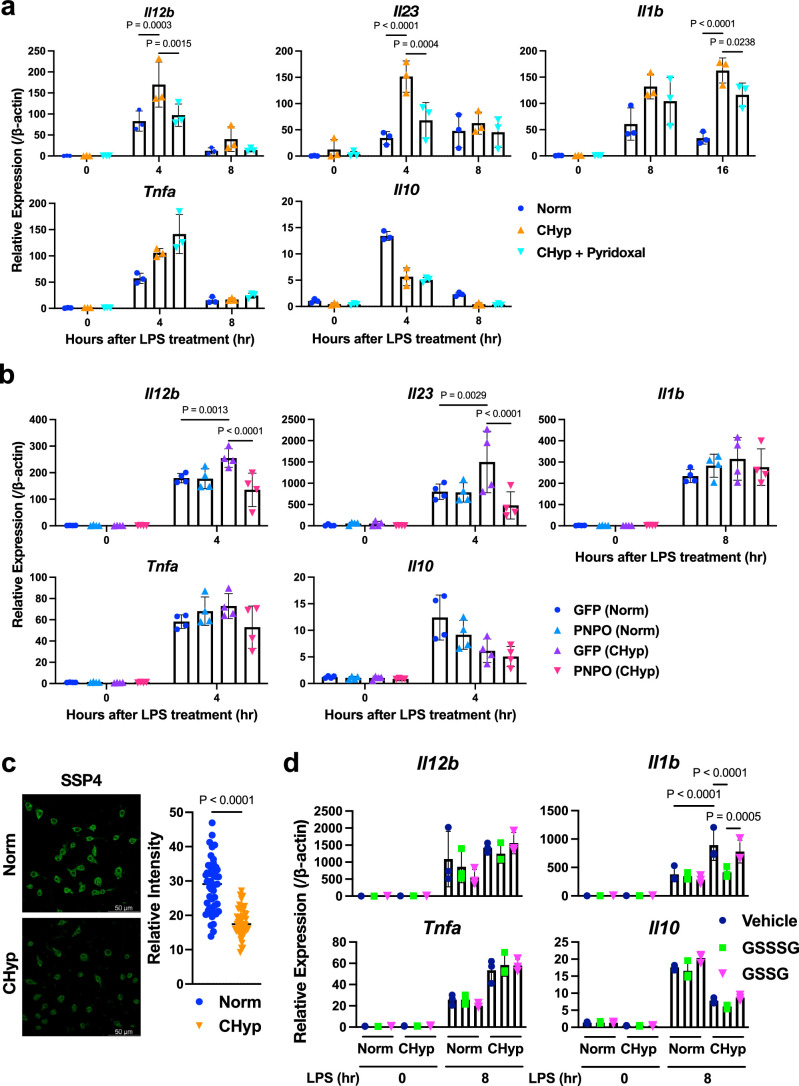
Supplementation of pyridoxal and supersulfide donor, GSSSG, to CHyp-BMDM. **a**. Expression of cytokine genes in BMDMs after LPS stimulation (n = 3). Norm: BMDMs differentiated and stimulated with LPS under normoxia, CHyp: BMDMs differentiated and stimulated with LPS under 1% O_2_, CHyp + Pyridoxal: BMDMs differentiated under 1% oxygen in the presence of 50 μg/ml pyridoxal. **b**. Expression of cytokine genes in BMDMs after LPS stimulation with or without PNPO overexpression (n = 3). GFP was overexpression as a negative control. **c**. SSP4 staining to detect supersulfides in BMDM. Representative SSP4 staining (left) and its quantification (right). Scale bars correspond to 50 μm. **d**. Expression of cytokine genes in BMDMs treated with a supersulfide donor, GSSSG, and oxidized glutathione, GSSG, as a negative control (n = 3). A representative result from 3 independent experiments is shown. Error bars represent S.E.M. (a, b, d). Two-way ANOVA (a, b, d) and two-sided Student’s *t*-test (c) were conducted to evaluate statistical significance. Source data

Because PLP is a biologically active form of vitamin B6 and functions as a cofactor for various enzymes, including transaminases and decarboxylases, we suspected that depletion of pyridoxal and PLP was one of the causes of lysosomal inhibition in CHyp BMDMs. To our delight, pyridoxal supplementation reversed the attenuated lysosomal acidification and the decreased Lamp1 protein under prolonged hypoxia (Fig. 7a,b). LPS-induced elevation of lysosome-related genes such as *Hck* and *Tlr3*, which was hampered in CHyp BMDMs, was also restored by pyridoxal (Fig. 7c). These data indicated that pyridoxal supplementation recovered lysosomal function in CHyp BMDMs. Consistent with these results, pyridoxal supplementation restored LPS-induced TET2 accumulation (Fig. 7d). Both pyridoxal supplementation and exogenous PNPO expression in CHyp BMDMs cancelled the overexpression of proinflammatory cytokine genes, especially *Il6*, *Il12b* and *Il23* (Fig. 7e–g and Extended Data Fig. 9a,b). These results indicate that prolonged hypoxia causes proinflammatory phenotypes of macrophages by attenuating the bioactivation of vitamin B6 that is required for lysosomal function. An exception was *Il10*, whose expression was neither reduced by *Pnpo* deficiency (Fig. 5f) nor recovered by pyridoxal supplementation or exogenous PNPO expression (Extended Data Fig. 9a,b), suggesting that the PNPO–PLP axis was not sufficient for the regulation of *Il10*.

We then examined how PLP regulates lysosomal activity. Based on recent studies showing anti-inflammatory activities of supersulfides^16–18^, which are synthesized by PLP-dependent enzymes, such as CSE, CBS, CARS1 and CARS2 (refs. ^14,15^), we hypothesized that decreased supersulfide synthesis causes the proinflammatory phenotypes of macrophages under chronic hypoxia. To evaluate this hypothesis, we measured supersulfide levels using SSP4, a fluorescent probe for supersulfide detection, and found that supersulfides were significantly decreased in CHyp BMDMs (Extended Data Fig. 9c), which was cancelled by pyridoxal supplementation (Fig. 7h). Supplementation with glutathione trisulfide (GSSSG), a supersulfide donor, but not oxidized glutathione (GSSG), increased lysosomal acidification and reduced the enhanced expression of proinflammatory cytokine genes, *Il6* and *Il1b*, in CHyp BMDMs (Fig. 7i,j and Extended Data Fig. 9d). These results suggest that impaired supersulfide synthesis due to PLP unavailability is a major cause of the macrophage phenotypes under chronic hypoxia.

### PNPO–PLP axis functions in mice under prolonged hypoxia

**Fig. 8:**
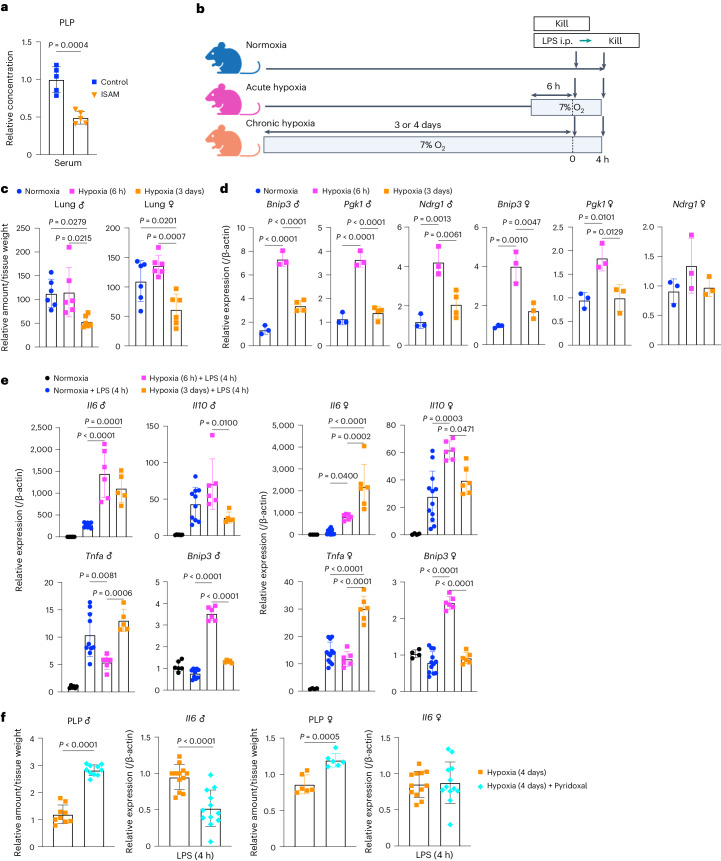
In vivo contribution of PNPO–PLP axis in prolonged hypoxia. **a**,**c**, Relative amount of PLP in serum of male ISAM and control mice (*n* = 5 mice for each group) (**a**) and lung tissues of mice exposed to acute (6 h) and chronic hypoxia (3 days) (*n* = 6 mice for each group) (**c**). **b**, Experimental design for exposure of mice to hypoxia. The illustration was created with BioRender.com. i.p., intraperitoneally. **d**, Expression of HIF target genes in lung tissues of mice exposed to acute (6 h) and chronic hypoxia (3 days) (*n* = 3 or 4 mice for each group). **e**, Gene expression at 4 h after LPS treatment in lung tissues (*n* = 5–10 for male, *n* = 4–12 for female). **f**, Pyridoxal supplementation to mice exposed to chronic hypoxia (4 days). Relative amount of PLP in lung tissues (*n* = 9–10 for male and *n* = 6 for female) and *Il6* expression at 4 h after LPS treatment in lung tissues (*n* = 12 for male and female) are shown. Error bars represent s.e.m. (**a**,**c**–**f**). A two-sided Student’s *t*-test (**a**,**f**) and one-way ANOVA (**c**–**e**) were conducted to evaluate statistical significance. Source data

PLP was decreased in the serum of a prolonged hypoxia mouse model ISAM, suggesting the functional significance of the PNPO–PLP axis in an in vivo context (Fig. 8a). To further evaluate the impact of PNPO–PLP axis on an alternative in vivo model, we exposed mice to 7% O_2_ for 6 h and 3 or 4 days, representing acute and chronic models, respectively (Fig. 8b). PLP levels were significantly reduced in the lung tissues of mice exposed to hypoxia for 3 days but not 6 h (Fig. 8c). Thus, the PLP reduction under prolonged hypoxia was observed not only in macrophages but also in vivo (also see Fig. 4c). In contrast, HIF target genes were highly upregulated after 6 h but not after 3 days of hypoxia (Fig. 8d), suggesting that HIF pathway activity is blunted during prolonged hypoxia. Thus, the HIF pathway mainly contributes to the acute phase of hypoxia and the PLP decrease becomes evident in the chronic phase of hypoxia.

**Extended Data Fig. 10:**
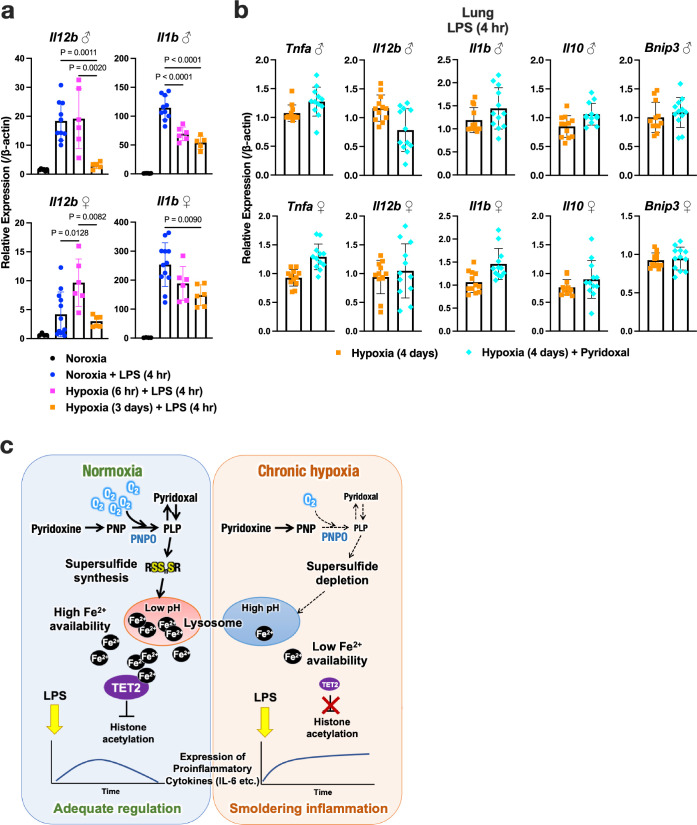
Metabolic oxygen sensing by PNPO-PLP axis controls lysosome activity and inflammatory response of macrophages. **a**. Gene expression at 4 hr after LPS treatment in lung tissues of mice exposed to acute (6 hr) and chronic hypoxia (3 days) (normoxia male, n = 6; normoxia+LPS male, n = 10; acute hypoxia + LPS male, n = 6; chronic hypoxia + LPS male, n = 5; normoxia female, n = 4; normoxia + LPS female, n = 12; acute hypoxia + LPS female, n = 6; chronic hypoxia + LPS female, n = 6). **b**. Gene expression at 4 hr after LPS treatment in lung tissues of mice exposed to chronic hypoxia (4 days) with or without pyridoxal supplementation (n = 12 for male and female). **c**. Schematic illustration of PNPO-PLP axis in response to chronic hypoxia. Prolonged hypoxic condition reduces PNPO activity and gradually decreases PLP that is required for the supersulfide synthesis and maintenance of lysosomal function. In macrophages, lysosomal inhibition caused by PLP insufficiency-induced supersulfide reduction limits Fe^2+^ availability and inhibits TET2 function, resulting in the delayed resolution of inflammation. Error bars represent S.E.M. (a, b). One-way ANOVA (a) and two-sided Student’s *t*-test (b) were conducted to evaluate statistical significance. None of the data shown in (b) reached statistical significance. Source data

We went on to examine the LPS-induced gene expression in lung tissues with or without hypoxia. *Bnip3* was upregulated after 6 h but not after 3 days of hypoxia as expected. Among the inflammatory cytokine genes examined, *Il6* was upregulated in hypoxia regardless of the duration compared to normoxia and *Tnfa* was upregulated only in prolonged hypoxia, although the difference did not reach statistical significance in male mice (Fig. 8e and Extended Data Fig. 10a). As PLP was decreased in chronic hypoxia, we examined whether PLP recovery could cancel the effects of chronic hypoxia on the cytokine gene expression. Pyridoxal was administered to the mice using an implantable osmotic pump, which successfully increased PLP levels in their lung tissues (Fig. 8f). When LPS was injected, the pyridoxal supplementation attenuated *Il6* expression in male mice but not in female mice (Fig. 8f), which may be because the pyridoxal administration was only modestly effective in increasing PLP in the female lung under prolonged hypoxia. This may be due to differences in the metabolic regulation of vitamin B6 between males and females. Expression levels of other cytokine genes, including *Tnfa*, and *Bnip3* were not changed by the pyridoxal administration (Extended Data Fig. 10b). The macrophage response to prolonged hypoxia was well recapitulated by *Il6* expression in lung tissue containing multiple cell lineages.

In summary, *Il6* upregulation in the acute hypoxia model, in which PLP levels were maintained, is considered independent of PNPO–PLP function. In contrast, *Il6* upregulation in the chronic hypoxia model, in which PLP levels and HIF activity were reduced, is considered to be a consequence of the PNPO–PLP response to chronic hypoxia independent of the HIF pathway, as pyridoxal supplementation reversed the phenotype.

## Discussion

Association between hypoxia and inflammation has been a subject of great interest and many studies have described that one of the key pathways involved in this connection is the PHD–HIF system by using short-term hypoxia^11–13^. In contrast, we applied long-term hypoxia to study the inflammatory response of macrophages and identified the PNPO–PLP axis as an HIF-independent oxygen-sensing mechanism that operates under prolonged hypoxia but not in the acute phase of hypoxia. An emerging concept from this work is ‘metabolic oxygen sensing’ in which prolonged hypoxia is sensed by PNPO, leading to the gradual decline of PLP-dependent metabolism and induction of a cellular state different from that induced in response to acute hypoxia, namely a lysosome-inhibited state (Extended Data Fig. 10d). For lysosomal regulation, supersulfide synthesis, which requires PLP, turned out to be a major downstream process of the PNPO–PLP axis. Notably, unbiased analyses led us to recognize the association between prolonged hypoxia, PLP decrease and lysosomal inhibition, underscoring the importance of this system in the biological context.

The *K*_m_ value of PNPO for O_2_ purified from rat liver has been reported to be 182 μM by using PNP as a substrate^29^. Although it is lower than those of PHD2 (250 μM)^2^ and KDM6A (200 μM)^3^, the *K*_m_ value of PNPO for O_2_ is almost comparable to that of KDM4A (173 μM)^30^, which is reported to be regulated by the oxygen concentration, suggesting that the oxygen concentration also regulates PNPO activity. Of note, congenital deficiency of PNPO shows early-onset neonatal encephalopathy that closely resembles hypoxic-ischaemic encephalopathy^31^. Based on these reports, we consider that PNPO is an oxygen sensor and persistent PNPO inhibition under prolonged hypoxia resulted in the depletion of PLP and pyridoxal.

Oxygen-sensing mechanisms known to date utilize dioxygenases with high *K*_m_ values for oxygen, namely PHD^2^, KDM6A^3^ and ADO^4^, as oxygen sensors for adaptation to low-oxygen conditions. These mechanisms primarily alter protein behaviour, such as protein stability and post-translational modification, which regulates gene expression and ultimately leads to metabolic changes. In contrast, the PNPO–PLP system primarily alters metabolite behaviour; vitamin B6 has been shown to be an oxygen-sensitive nutrient that regulates PLP-dependent metabolism and induces metabolic rewiring during prolonged hypoxia, ultimately leading to transcriptional and epigenetic regulation mediated by, for example, TET2 in macrophage.

Supersulfides were found to be an important mediator connecting the PNPO–PLP axis and lysosome. Although how supersulfides maintain the lysosomal acidification still remains unknown, at least two mechanistic explanations are possible based on the previous reports describing that the p*K*_a_ value of hydropersulfide is lower than that of hydrosulfide^32^ and that protein persulfidation alters and regulates protein function^33^. One possibility is that supersulfides, namely hydropersulfides and hydropolysulfides, act directly as acids to lower lysosomal pH and the other is that supersulfidation of proteins involved in maintaining lysosomal pH is required for their proper function. In this study, the enzymes responsible for the supersulfide synthesis were not determined because each of the currently known supersulfide-synthesizing enzymes, CBS, CSE, CARS1 and CARS2, possesses different additional enzymatic activities, making it unpreferable in terms of specificity to knock out and overexpress the genes encoding these enzymes. Rather, we propose that PLP abundance can regulate the entire battery of supersulfide-synthesizing enzymes and that PLP serves as a new regulatory layer for the supersulfide production.

In macrophages, *Il6* and other proinflammatory cytokine genes were upregulated by prolonged hypoxia and by *Pnpo* deficiency and their enhanced expression in hypoxia was reversed by reactivation of the PNPO–PLP axis, confirming that the PNPO–PLP axis regulates a battery of proinflammatory cytokine genes in prolonged hypoxia. In the mouse lung, *Il6* was the only gene that was upregulated by prolonged hypoxia and whose increased expression was reversed by PLP. This difference is likely due to the multiple cell lineages present in the lung. According to the recent single cell analyses of human and mouse lung tissue, *Il6* is broadly expressed in various cell types such as fibroblasts and endothelial cells in addition to macrophages, whereas other cytokine genes are mainly expressed in macrophages (https://www.proteinatlas.org/; https://panglaodb.se/index.html). We speculate that cytokine genes expressed in the limited cell population in the lung were not properly quantified in the whole lung measurement. On the other hand, the similarity of *Il6* expression patterns in the whole lung and macrophages strongly suggests that *Il6* is regulated by the PNPO–PLP axis during prolonged hypoxia not only in macrophages but also in other cell lineages in the lung.

PLP insufficiency has been shown to correlate with inflammation status^34,35^ and therapeutic potential of PLP for various inflammatory diseases has been suggested^36,37^. These previous studies are consistent with our results that prolonged hypoxia inhibits bioactivation of vitamin B6 and predisposes to exacerbated inflammation. While we focused on lysosomal function in prolonged hypoxia as an action target of PLP, PLP was also reported to suppress inflammasome activation^38^. As lysosomal dysfunction has been shown to cause excessive inflammasome activation^39^, we speculate that restoration of lysosomal integrity by PLP may limit the inflammasome activation in macrophages under prolonged hypoxia in addition to the recovery of TET2-mediated transcriptional regulation for inflammation resolution.

Recent studies have shown that lysosomes are a dynamic structure that mediates the adaptation of cell metabolism to environmental cues^40^. Inflammation is one of the major consequences of lysosomal inhibition^41,42^, which may explain proinflammatory tendencies of patients suffering from systemic chronic hypoxia. Alternatively, restraining lysosomal activity preserves the quiescence and potency of haematopoietic stem cells^43^. Considering the local hypoxic environment of the bone marrow niche where the haematopoietic stem cells reside, limited production of PLP may contribute to the inhibition of lysosomal activity for the preservation of haematopoietic stem cells. Lysosomal inhibition by the PNPO–PLP axis under chronic hypoxia is likely to underlie various pathological and physiological processes.

## Methods

### Mice

Male ISAM and their control mice^19,20^ were used for experiments at 4–5 months after birth because males exhibit systemic hypoxia with less individual variation than females. Blood was drawn from anaesthetized mice using heparinized capillary tubes (Fisher Scientific) into the microtube within 0.5 M EDTA (pH 7.4, 2 μl) and centrifuged (1,000*g* for 15 min at 4 °C) to isolate serum for metabolome analysis. For exposure to prolonged hypoxia, C57BL/6N male and female mice were used at 2–3 months after birth.

For preparation of BMDMs, wild-type mice, *Hif1a*^F/F^ mice (*Hif1a* Ctrl), *Hif1a*^F/F^:Tie2-Cre mice (*Hif1a* KO), *Vhl*^F/F^ mice (*Vhl* Ctrl) and *Vhl*^F/F^:Lys-Cre mice (*Vhl* KO), which were all on a C57BL/6 background, were used at 2–4 months after birth. *Hif2a*^F/F^ mice (*Hif2a* Ctrl), *Hif2a*^F/F^:ROSA-Cre^ERT2^ mice (*Hif2a* KO), *Pnpo*^F/F^ mice (*Pnpo* Ctrl) and *Pnpo*^F/F^:ROSA-Cre^ERT2^ mice (*Pnpo* KO), which were on a mixed background, were treated with tamoxifen (daily i.p. injection for 1 week) at 2–4 months after birth to induce Cre recombinase activity and used for BMDM preparation 1 week after the last tamoxifen treatment. Because BMDMs cultured from male and female mice gave substantially the same results, data from male and female BMDMs were combined and presented in a single figure.

*Hif1a*^F/F^ mice (B6.129-Hif1a^tm3Rsjo^/J), *Hif2a*^F/F^ mice (Epas^tm1Mcs^/J), *Vhl*^F/F^ mice (B6.129S4(C)-Vhl^tm1Jae^/J), Tie2-Cre mice (B6.Cg-Tg(Tek-cre)12Flv/J), Lys-Cre mice (B6.129P2-Lyz2^tm1(cre)Ifo^/J) and ROSA-Cre^ERT2^ mice (B6.129-Gt(ROSA)26Sor^tm1(cre/ERT2)Tyj^/J) were purchased from the Jackson Laboratory. *Pnpo*^F/F^ mice were newly established by inserting loxP sequences into the first and second introns of the *Pnpo* gene by a CRISPR-Cas9 genome editing method in the Laboratory Animal Resource Center at the University of Tsukuba.

These mice were housed in a specific-pathogen-free facility and maintained under constant temperature (24 °C), humidity (40%) and 12-h light–dark cycle, with food and water provided ad libitum according to the regulations of the Standards for Human Care and Use of Laboratory Animals of Tohoku University (Tohoku University, 2007), the Guidelines of Jichi Medical University upon approval of the Use and Care of Experimental Animals Committee of Jichi Medical University and the Guidelines for Proper Conduct of Animal Experiments by the Ministry of Education, Culture, Sports, Science and Technology of Japan (Science Council of Japan, 2006). The animal experiments were performed according to the protocol 2021AcA-002 approved by the Tohoku University Animal Care and Use Committee.

### Evaluation of tissue hypoxia in ISAM

To detect tissue hypoxia, male ISAM and control mice were i.p. injected with 60 mg kg^−1^ pimonidazole (Hypoxyprobe) and killed 1 h after the injection. The liver, kidney and intestines were used for immunoblotting and the intestines were also used for immunohistochemistry.

For immunoblotting, the intestines were lysed with RIPA buffer (50 mM Tris-HCl, pH 8.0, 150 mM NaCl, 0.5% sodium deoxycholate, 0.1% SDS and 1.0% NP-40) containing 10 µM MG132 and 1.0% protease inhibitor cocktail (Nacalai Tesque). The lysates were separated by SDS–PAGE and transferred onto a PVDF membrane. The membranes were reacted with antibodies against pimonidazole (Pab2627, Hypoxyprobe, 1:1,000 dilution) and β-tubulin (605102, BioLegend, 1:10,000 dilution).

For immunohistochemistry, 3-μm paraffin sections were used. Paraffin-embedded samples were antigen unmasked by target retrieval solution (Dako; pH 6.0). Sections were blocked and incubated with a primary antibody against pimonidazole (Pab2627, Hypoxyprobe, 1:50 dilution). Signals were obtained using diaminobenzidine solution (Dako), and sections were counter stained by hematoxylin.

### DSS-induced colitis model

Age-matched mice (5–6 months) received 3% DSS (MP Biomedicals) in drinking water ad libitum for 6 days to induce colitis. After the mice were killed by cervical dislocation, the colon was subsequently dissected for analysis. Samples were fixed in Mildform 10N (Wako) at 4 °C overnight and processed for paraffin block preparation or frozen in liquid nitrogen and stored at −80 °C until gene expression analysis.

### Histological analysis of DSS-treated mouse colons

Paraffin sections of colons were stained with haematoxylin and eosin. Pathological alterations were analysed and scored following the criteria published previously^44^. In brief, inflammatory cell infiltration and changes in intestinal architecture were rated on a scale of 0 to 3 for each. A total score was on a scale of 0 to 6 per field. Three fields per sample, each one from the proximal, intermediate and distal colon, were independently evaluated and the scores were summed to determine the pathological score.

### Preparation of peritoneal exudate macrophages

For the collection of peritoneal exudate macrophages, mice were i.p. injected with 2 ml 4% thioglycolate. Peritoneal cells were isolated from exudates in the peritoneal cavity 3 days after the injection, incubated for 2 h in cell culture plates and washed with PBS. The adherent cells were used for experiments.

### Preparation of BMDMs

Bone marrow cells were flushed into PBS containing 3% fetal bovine serum (FBS) and passed through a 70-μm nylon mech cell strainer (Falcon). The obtained whole bone marrow cells were collected by centrifugation at 800 rpm for 5 min. The cell pellet was resuspended in red cell lysis buffer (150 mM NH_4_Cl, 1 mM KHCO_3_, 0.1 mM Na_2_EDTA and 10 mM phosphate buffer) and incubated on ice for 20 min to lyse erythrocytes. After another centrifugation at 800 rpm for 5 min, cells were washed in Dulbecco’s modified Eagle’s medium (DMEM) (low-glucose with l-glutamine) once and seeded in DMEM supplemented with 40 ng ml^−1^ M-CSF (PeproTech), 10% FBS and penicillin/streptomycin. The cells were cultured at 37 °C under 5% CO_2_ and saturated humidity in 1% O_2_, 5% O_2_ or normoxia. After 1 week, the culture medium was replaced with fresh DMEM supplemented with 10% FBS and penicillin/streptomycin without M-CSF.

### Evaluation of Hif2a disruption efficiency

Genomic DNA was purified from BMDMs, which were cultured from *Hif2a*^F/F^ mice and *Hif2a*^F/F^:ROSA-Cre^ERT2^ mice after tamoxifen treatment. Genotyping PCR was conducted according to a previous report^45^. A genomic region containing the exon 2 of *Hif2a* gene (floxed allele) was amplified with a primer set, Hif2a-P1: 5′-CAG GCA GTA TGC CTG GCT AAT TCC AGT T-3′ and Hif2a-P2: 5′-CTT CTT CCA TCA TCT GGG ATC TGG GAC T-3′. A genomic region without the exon 2 of *Hif2a* gene (deleted allele) was amplified with a primer set, Hif2a-P1: 5′-CAG GCA GTA TGC CTG GCT AAT TCC AGT T-3′ and Hif2a-P3: 5′-GCT AAC ACT GTA CTG TCT GAA AGA GTA GC-3′.

### Reagents

The following 2OG-dependent dioxygenase inhibitors were used; KDM6A inhibitor, GSKJ4 (4594, TOCRIS Bioscience), KDM5A inhibitor, KDM5-C70 (M60192-2s, Xcess Biosciences), TET inhibitor, Bobcat339 (V4331, InvivoChem) and PHD inhibitor, GSK360A (G797600, Toronto Research Chemicals). The following lysosomal inhibitors were used; ConA (BVT-0237-M001, AdipoGen Life Sciences) and Baf (BVT-0252-M001, AdipoGen Life Sciences). GSSG was purchased from FUJIFILM Wako. GSSSG was synthesized as previously described^46^. Both GSSSG and GSSG were dissolved in distilled water at a concentration of 10 mM at the time of use.

### LPS treatment of macrophages

BMDMs were stimulated with 100 ng ml^−1^ LPS from *Escherichia* *coli* 0111:B4 (Sigma-Aldrich) 1 day after the medium change. For the normoxia control, BMDMs were differentiated in normoxia and continuously cultured in normoxia after LPS stimulation (Norm BMDMs). For the assay of chronic hypoxia, BMDMs were differentiated in 1% O_2_ and continuously cultured in 1% O_2_ after LPS stimulation (CHyp BMDMs). For the assay of acute hypoxia, BMDMs were differentiated in normoxia, incubated in 1% O_2_ for 12 h before LPS stimulation, and cultured in 1% O_2_ after LPS stimulation (AHyp BMDMs). BMDMs were collected for RNA purification, immunoblot analysis and iron and lysosomal staining at the indicated time points.

For pretreatment with lysosomal inhibitors, BMDMs differentiated under normoxia were treated with 10 nM ConA, 10 nM Baf or vehicle (DMSO) at 16 h before LPS stimulation. For pretreatment with inhibitors of 2OG-dependent dioxygenases, BMDMs were differentiated under normoxia in the presence of the 2OG-dependent dioxygenase inhibitors or vehicle (DMSO) and stimulated with LPS under normoxia. Then, 2 μM GSKJ4 for KDM6A inhibition, 1 μM KDM5-C70 for KDM5A inhibition, 100 μM Bobcat339 for TET inhibition and 5 μM GSK360A for PHD inhibition were used. For pretreatment with pyridoxal, BMDMs were differentiated under 1% O_2_ in the presence of 50 μg ml^−1^ pyridoxal. For pretreatment with GSSSG and GSSG, BMDMs were differentiated under normoxia or 1% O_2_ in the presence of 5 μM GSSSG or GSSG.

### RNA-seq analysis

For RNA-seq analysis of BMDMs cultured under different oxygen tension, total RNA was extracted from BMDMs using the RNeasy Mini kit (QIAGEN) in biological duplicates. Total RNA from the BMDMs was used to prepare complementary DNA sequencing libraries using the SureSelect Strand-Specific RNA library preparation kit (Agilent Technologies) after the poly-A selection step. The libraries were sequenced on a HiSeq 2500 sequencing system (Illumina), generating 76-base single-end reads. Raw fastq sequencing files were analysed by FastQC v.0.11.5 (http://www.bioinformatics.babraham.ac.uk/projects/fastqc/) to check their sequence quality and possible adapters, poly-A tails and low-sequence quality bases were trimmed by Cutadapt v.1.15 (ref. ^47^). After trimming, all the remaining reads were aligned to the mm9 reference genome using STAR v.2.5.3a^48^ with the primary genome annotation in GENCODE Release M1 (ref. ^49^). After mapping, Cuffquant and Cuffnorm software, part of the Cufflinks suite v.2.2.1 (ref. ^50^), were used to calculate an FPKM (fragments per kilobase of transcript sequence per million mapped fragments) value for each gene.

For RNA-seq analysis of BMDMs treated with lysosomal inhibitors, total RNA was extracted from BMDMs using the RNeasy Mini kit (QIAGEN) in biological duplicates. Total RNA from the BMDMs was used to prepare cDNA sequencing libraries using the TruSeq Stranded mRNA Library Prep kit (Illumina). The libraries were sequenced on a NovaSeq 6000 sequencing system (Illumina), generating 101-bp-end reads. Raw fastq sequencing files were analysed as described above.

### Informatic analysis of RNA-seq data

Scatter-plots and heatmaps were visualized by Rstudio packages ggplot2 and pheatmap, respectively. Enrichment analysis was performed using a browser platform of Enrichr (https://maayanlab.cloud/Enrichr/). Multiple comparison was performed within the program. Downloaded result files were visualized as dotplots using ggplot2. Rstudio was utilized under Rstudio v.2021.09.1+372. GSEA was conducted using a browser platform GSEA v.4.1.0. with gene_set permutation and default parameters.

For generating scatter-plots showing correlations among impacts of hypoxia and lysosomal inhibition on LPS-induced transcriptome, we first approximated total amount of transcripts over time as the AUC calculated from mRNA levels at each time point from 0 to 24 h after LPS addition. AUC ratios of CHyp BMDMs versus Norm BMDMs and AHyp BMDMs versus Norm BMDMs were plotted for each gene to compare effects of chronic and acute hypoxia. AUC ratios of ConA-treated versus DMSO-treated BMDMs and Baf-treated BMDMs versus DMSO-treated BMDMs were plotted for each gene to compare effects of ConA and Baf. AUC ratios of ConA-treated BMDMs versus DMSO-treated BMDMs and CHyp BMDMs versu Norm BMDMs were plotted for each gene to compare effects of ConA and chronic hypoxia.

Gene sets for GSEA were defined as follows. ConA_up and ConA_down: upregulated and downregulated genes by more than fourfold (log_2_ 4) by ConA treatment, respectively. Baf_up and Baf_down: upregulated and downregulated genes by more than fourfold (log_2_ 4) by Baf treatment, respectively. CHyp_up and CHyp_down: upregulated and downregulated genes by more than fourfold (log_2_ 4) by chronic hypoxia.

### Cell culture

U937 cells were cultured and maintained in DMEM containing 10% FBS (Biosera) under 5% CO_2_ at 37 °C. U937 cells were used after differentiation into macrophage-like cells by the treatment with 10 ng ml^−1^ PMA for 3 days. HeLa cells (purchased from RIKEN, RCB3680) were cultured and maintained in DMEM containing 10% FBS (Biosera) under 5% CO_2_ at 37 °C.

### Transfection of siRNA

U937 cells was transfected with siRNAs by using GenomONE-Si (Ishihara Sangyo) according to the manufacturer’s protocol. PMA was added right after the transfection. The cells were analysed 72 h after the transfection. Control (MISSION siRNA Universal Negative Control #1, Sigma), *PNPO* si1 (SASI_Hs01_00068079, Sigma) and *PNPO* si2 (SASI_Hs02_00351341, Sigma) were used. To prepare the cells for PLP measurement, U937 cells treated with siRNA and PMA were incubated in 1% O_2_ for 72 h. After being washed in PBS once, the cells were reoxygenated in normoxia-equilibrated and prewarmed medium containing 4.0 mg l^−1^ pyridoxine hydrochloride.

HeLa cells were transfected with siRNAs by using Lipofectamine RNAiMAX (Thermo Fisher Scientific) according to the manufacturer’s protocol. Control (MISSION siRNA Universal Negative Control #1, Sigma), *ARNT* si1 (SASI_Hs01_00167000, Sigma) and *ARNT* si2 (SASI_Hs02_00167001, Sigma) were used.

### ChIP assay

ChIP assays were performed with BMDMs differentiated in 1% O_2_ and normoxia using anti-H3K27ac antibody (MABI0309, MAB Institute) and anti-H3K4me3 antibody (MABI0304, MAB Institute). The cells were treated with 100 ng ml^−1^ LPS for 12 h and cross-linked with 1% formaldehyde for 10 min. The samples were then lysed and sonicated to shear DNA. Sonication was conducted according to previously described procedures^51,52^. The solubilized chromatin fraction was incubated overnight with anti-H3K27ac antibody or anti-H3K4me3 antibody that was prebound to Dynabeads anti-rabbit IgG (Life Technologies). Then, 0.2 μg anti-H3K27ac antibody and 2 μg anti-H3K4me3 antibody were used per sample collected from one 10-cm dish. Precipitated DNA was analysed by real-time PCR. The primer sets used in the ChIP assay are listed in Supplementary Table 1.

### ELISA

PMs or BMDMs at 2 × 10^6^ cells per ml were incubated with 100 ng ml^−1^ LPS for 12 h or 24 h to measure TNF-α and IL-6 and further incubated for 2 h with 1 mM ATP to measure IL-1β. The culture supernatants were assessed for the cytokines using mouse TNF-α, IL-6 and IL-1β ELISA kits (R&D Systems).

### RNA purification and quantitative RT–PCR

Total RNA samples were prepared from cells and tissues using ISOGEN (Nippon Gene) or ReliaPrep RNA Miniprep Systems (Promega) according to the manufacturer’s instructions. First-strand cDNA was synthesized from 100 ng of total RNA using ReverTra Ace qPCR RT Master Mix with gDNA Remover (TOYOBO). Real-time PCR was performed in triplicate for each sample with QuantStudio real-time PCR system (Thermo Fisher Scientific) using KAPA SYBR FAST qPCR Master Mix (Kapa Biosystems). Expression levels of *Actb* (β-actin) gene were used as internal controls for normalization. The primer sets used in the real-time PCR are listed in Supplementary Table 2.

### Immunoblot analysis

Immunoblot analyses were performed as described previously^53^. The antibodies that were used were anti-TET2 (ab124297, Abcam, 1:2,000 dilution), KDM5a (ab70892, Abcam, 1:2,000 dilution), anti-KDM6a (33510, Cell Signalling Technology, 1:2,000 dilution), anti-PHD2 (NB100-2219, Novus, 1:2,000 dilution), anti-Lamp1 (ab25245, Abcam, 1:2,000 dilution), anti-PNPO (15552-1-AP, Proteintech, 1:5,000 dilution), anti-HIF-2α (ab109616, Abcam, 1:2,000), anti-p70 S6K (2708, Cell Signalling Technology, 1:5,000 dilution), anti-phospho-p70 S6K (9234, Cell Signalling Technology, 1:5,000 dilution) and anti-tubulin (T9026, Sigma, 1:10,000 dilution).

### Detection of lysosomal acidification

Lysosomal acidity was observed by using an AcidiFluor ORANGE kit (Goryo Chemical) according to the manufacturer’s instructions. Nuclei were imaged with Hoechst 33342 (Dojindo). In brief, BMDMs, U937 and HeLa cells were prepared in four-chamber 35-mm culture dishes at a density of 2 × 10^5^ cells per well under the indicated conditions. The cells were stained with 2 μM AcidiFluor ORANGE and 0.5 μg ml^−1^ Hoechst 33342 for 2 h at 37 °C under 5% CO_2_. Then, the cells were washed three times with PBS and observed with confocal microscopy (TCS SP8, Leica). AcidiFluor ORANGE was detected at Ex/Em = 552/570–590 nm. The fluorescence intensities were quantified by using LASX software (Leica). A single cell was circled and the intensity of each circle was quantified. The AcidiFluor ORANGE intensity was normalized by Hoechst 33342 intensity for each cell.

### Detection of intracellular ferrous iron

The iron concentration was assessed using FerroOrange (DojinDo) to measure intracellular ferrous iron levels according to the manufacturer’s protocol. In brief, BMDMs were prepared in four-chamber 35-mm culture dishes at a density of 2 × 10^5^ cells per well and treated with or without LPS for 4 h. The cells were washed three times with PBS and stained with FerroOrange working solution for 30 min at 37 °C under 5% CO_2_. Then, the cells were observed with confocal microscopy (TCS SP8, Leica) at Ex/Em = 552/561–570 nm. The fluorescence intensity was measured by ImageJ software (National Institutes of Health). Seven fields per sample were independently evaluated.

### Treatment with ferric ammonium citrate

BMDMs differentiated under normoxia were treated with 10 nM ConA or vehicle (DMSO) with or without 0.1 mg ml^−1^ Fe at 16 h before LPS stimulation. Then cells were collected for ferrous iron detection, gene expression analysis and immunoblot analysis.

### Detection of intracellular supersulfides

Intracellular supersulfides were detected using Sulfane Sulfur Probe 4 (SSP4, Dojindo) as previously described^54^. In brief, BMDMs were seeded in four-chamber 35-mm culture dishes at a density of 1 × 10^5^ cells per well and incubated for designated periods of time. After two washes with serum-free low-glucose DMEM, 20 μM SSP4 working solution containing 0.5 mM cetyltrimethylammonium bromide was added to the cells and incubated at 37 °C and 5% CO_2_ for 15 min. Then, the cells were washed twice with PBS and observed at Ex/Em = 488/515–535 nm with confocal microscopy (TCS SP8, Leica). The fluorescence intensity was measured by ImageJ (National Institutes of Health).

### Methylome analysis

Methylome data were obtained with whole-genome bisulfite sequencing and libraries were prepared based on post-bisulfite adaptor tagging)^55^ using an improved protocol tPBAT^56^. A total of 150 ng purified genomic DNA was used for each sample. PCR amplification was not performed for any library. After the preparation, libraries of three biological replicates tagged with different indices were mixed equally. The mixture was sequenced using the HiSeq X ten system. Sequencing was performed by Macrogen. The obtained reads were mapped to the reference genome and summarized with an in-house pipeline^55^.

### Metabolome analysis

Total metabolites were extracted from BMDMs and mouse serum according to the Bligh and Dyer method with minor modifications^57^. For metabolite measurement in BMDMs, BMDMs were washed twice with cold PBS and collected in 1 ml cold methanol (−30 °C) containing 10-camphorsulfonic acid (1.5 nmol) and piperazine-1,4-bis(2-ethanesulfonic acid) (PIPES) (1.5 nmol) as internal standards. The samples were vigorously mixed by vortexing for 1 min followed by 5 min of sonication. To precipitate protein, the methanol extracts were incubated on ice for 5 min. The extracts were then centrifuged at 16,000*g* for 5 min at 4 °C, and the resultant supernatant was collected. Protein concentrations in the pellet were determined using a Pierce BCA Protein Assay kit (Thermo Fisher Scientific). Then, 600 μl supernatant was transferred to another tube and 600 μl chloroform and 480 μl water were added. After centrifugation at 16,000*g* and 4 °C for 5 min, 800 μl of the upper layer was isolated and used for hydrophilic metabolite analysis.

For metabolite measurement in mouse serum, 50 μl serum was mixed with 940 μl methanol and 10 μl internal standard solution containing 10-camphorsulfonic acid (2.0 nmol) and PIPES (2.0 nmol). The samples were centrifuged at 16,000*g* at 4 °C for 5 min, and the supernatant (400 μl) was collected in clean tubes. After mixing with 400 μl chloroform and 320 μl water, phase separation of aqueous and organic layers was performed via centrifugation (16,000*g*, 4 °C, 5 min). The aqueous (upper) layer (500 μl) was transferred into a clean tube. After the aqueous layer extracts were evaporated under vacuum, the dried extracts were stored at −80 °C until the analysis of hydrophilic metabolites. Before analysis, the dried aqueous layer was reconstituted in 50 μl water.

Anionic polar metabolites (for example, succinate, lactate and PLP) were analysed via ion chromatography (Dionex ICS-5000+ HPIC system, Thermo Fisher Scientific) with a Dionex IonPac AG11-HC-4-μm guard column (2 mm internal diameter (i.d.) × 50 mm, 4-μm particle size, Thermo Fisher Scientific) and a Dionex IonPac AS11-HC-4-μm column (2 mm i.d. × 250 mm, 4-μm particle size, Thermo Fisher Scientific) coupled with a Q Exactive, high-performance benchtop quadrupole Orbitrap high-resolution tandem mass spectrometer (Thermo Fisher Scientific) (IC/HRMS/MS)^58^. Cationic polar metabolites (for example, pyridoxal, pyridoxine) were analysed via liquid chromatography (Nexera X2 UHPLC system, Shimadzu) with a Discovery HS F5 column (2.1 mm i.d. × 150 mm, 3-μm particle size, Merck) coupled with a Q Exactive instrument (PFPP-LC/HRMS/MS)^47^.

### Informatic analysis of metabolome data

Metabolome data obtained from BMDMs differentiated in 1% O_2_ and normoxia irrespective of the LPS treatment were used for the creation of a volcano plot. A Welch’s *t-*test was conducted for statistical significance.

### Quantification of PLP

PLP was quantified using VB6 Enzymatic assay kit (Bühlmann) according to the manufacture’s protocol. In brief, BMDMs, U937 and HeLa cells cultured in 24-well plates were washed with PBS once and then collected in 100 μl methanol. The methanol extracts were diluted by ‘substrate buffer’ for the measurement. Lung tissues were weighed and homogenized in methanol at the ratio of 100 mg tissue per ml methanol. After centrifugation at 5,000*g* for 5 min, the supernatant was diluted by 500-fold and added to the ‘substrate buffer’ for the measurement.

### Pyridoxine restriction culture of BMDM and U937 cells

Pyridoxine-depleted DMEM was manufactured on request (Cell Science & Technology Institute). For experiments using BMDM, the pyridoxine-depleted DMEM was used from the beginning of the BMDM differentiation. For experiments using U937, normal DMEM was changed to the pyridoxine-depleted DMEM at the time of PMA addition.

### PNPO overexpression in BMDM

Mouse PNPO-expressing adenovirus vector (pAV-EGFP-CMV-mPNPO) and GFP-expressing adenovirus vector (pAV-CMV-EGFP) were constructed and packaged into adenovirus particles in VectorBuilder. The adenovirus particles were added to the M-CSF-containing differentiation medium of BMDM 24 h before the medium change. The adenovirus particles were removed at the time of the medium change.

### Hypoxia exposure and LPS treatment of mice

A hypoxia chamber, in which the oxygen concentration was regulated by an oxygen controller (ProOx; BioSpherix) with a nitrogen generator (Nilox; Sanyo Electronic Industries), was used to expose the mice to hypoxic conditions. To avoid accumulation of moisture and carbon dioxide, a moisture absorber and Litholyme were set in the chamber, respectively. To reduce ammonia during the hypoxia exposure, cat litter was layered beneath the wooden chip bedding of mouse cages. Male and female mice were exposed to 7% O_2_ for 6 h or 3 days and killed for PLP measurement. For LPS treatment, the mice were exposed to 7% O_2_ for 6 h or 3 days and given an i.p. injection of LPS at a dose of 5 mg kg^−1^ body weight. The mice were killed at 4 h after the LPS injection. For supplementation of pyridoxal, ALZET osmotic pump 1007D containing 150 mg ml^−1^ pyridoxal hydrochloride (P9130, Sigma-Aldrich) in PBS was subcutaneously implanted to the mice under general anaesthesia and the exposure to 7% O_2_ was started from the next day. Control mice underwent sham operation of the back skin under general anaesthesia and were similarly exposed to the hypoxia. After a 4-day exposure to 7% O_2_, the mice were injected with LPS (5 mg kg^−1^ body weight) and killed 4 h after the treatment. Wild-type C57BL/6N male and female mice were randomly divided into the required number of groups for each experiment.

### Blinding

Histological evaluation of mouse intestine samples was blindly performed. In other experiments, data collection and analysis were not performed blind to the conditions of the experiments.

### Statistics and reproducibility

Throughout this study, no animals or data points were excluded. Statical analysis was conducted using Prism v.7 (GraphPad Software). Data are presented as mean ± s.e.m. or s.d. and were tested with an unpaired Student’s *t-*test, Welch’s *t-*test and one-way or two-way analysis of variance (ANOVA) followed by Tukey’s multiple comparison test. A *P* value <0.05 was considered significant. Data distribution was assumed to be normal, but this was not formally tested. Instead, we showed data distribution by indicating individual data points. No statistical methods were used to predetermine sample sizes, but our sample sizes are similar to those reported in previous publications^17,18^.

### Reporting summary

Further information on research design is available in the Nature Portfolio Reporting Summary linked to this article.

## Supplementary information

Reporting Summary

Supplementary Table 1 Supplementary Table 1: primers used for ChIP assay.

Supplementary Table 2 Supplementary Table 2: primers used for RT–PCR.

Supplementary Table 3 Supplementary Table 3: metabolites detected in metabolome analysis.

## Source data

Source Data Fig. 1 Statistical source data.

Source Data Fig. 2 Statistical source data.

Source Data Fig. 3 Statistical source data.

Source Data Fig. 4 Statistical source data.

Source Data Fig. 5 Statistical source data.

Source Data Fig. 6 Statistical source data.

Source Data Fig. 7 Statistical source data.

Source Data Fig. 8 Statistical source data.

Source Data Extended Data Fig. 1 Statistical source data.

Source Data Extended Data Fig. 2 Statistical source data.

Source Data Extended Data Fig. 4 Statistical source data.

Source Data Extended Data Fig. 5 Statistical source data.

Source Data Extended Data Fig. 6 Statistical source data.

Source Data Extended Data Fig. 7 Statistical source data.

Source Data Extended Data Fig. 8 Statistical source data.

Source Data Extended Data Fig. 9 Statistical source data.

Source Data Extended Data Fig. 10 Statistical source data.

Source Data Unprocessed Blots Unprocessed blots and gels for Figs. 3, 5–7 and Extended Data Figs. 1, 2 and 5.

## Data Availability

RNA-seq data were deposited at the Gene Expression Omnibus under accession codes GSE181641, GSE181642 and GSE181643. Metabolome data are shown in Supplementary Table 3. The mm9 reference genome data are available at https://www.gencodegenes.org/mouse/release_M1.html. Source data are provided with this paper.
