# Author Correction: PNPO–PLP axis senses prolonged hypoxia in macrophages by regulating lysosomal activity

**DOI:** 10.1038/s42255-024-01183-9

**Published:** 2024-11-26

**Authors:** Hiroki Sekine, Haruna Takeda, Norihiko Takeda, Akihiro Kishino, Hayato Anzawa, Takayuki Isagawa, Nao Ohta, Shohei Murakami, Hideya Iwaki, Nobufumi Kato, Shu Kimura, Zun Liu, Koichiro Kato, Fumiki Katsuoka, Masayuki Yamamoto, Fumihito Miura, Takashi Ito, Masatomo Takahashi, Yoshihiro Izumi, Hiroyuki Fujita, Hitoshi Yamagata, Takeshi Bamba, Takaaki Akaike, Norio Suzuki, Kengo Kinoshita, Hozumi Motohashi

**Affiliations:** 1 Department of Medical Biochemistry, Tohoku University Graduate School of Medicine, Sendai, Japan; 2 Division of Cardiology and Metabolism, Center for Molecular Medicine, Jichi Medical University, Shimotsuke, Japan; 3 Department of Cardiovascular Medicine, Graduate School of Medicine, The University of Tokyo, Tokyo, Japan; 4 Department of Gene Expression Regulation, IDAC, Tohoku University, Sendai, Japan; 5 Department of System Bioinformatics, Graduate School of Information Sciences, Tohoku University, Sendai, Japan; 6 Department of Integrative Genomics, Tohoku Medical Megabank Organization, Tohoku University, Sendai, Japan; 7 Data Science Center, Jichi Medical University, Shimotsuke, Japan; 8 Division of Oxygen Biology, United Centers for Advanced Research and Translational Medicine, Tohoku University Graduate School of Medicine, Sendai, Japan; 9 Department of Biochemistry and Molecular Biology, Tohoku Medical Megabank Organization, Tohoku University, Sendai, Japan; 10 Department of Biochemistry, Kyushu University Graduate School of Medical Sciences, Fukuoka, Japan; 11 Division of Metabolomics, Medical Institute of Bioregulation, Kyushu University, Fukuoka, Japan; 12 Advanced Research Laboratory, Canon Medical Systems Corporation, Otawara, Japan; 13 Department of Environmental Medicine and Molecular Toxicology, Tohoku University Graduate School of Medicine, Sendai, Japan

**Keywords:** Metabolomics, Lysosomes, Inflammation, Metabolism, Monocytes and macrophages

Correction to: *Nature Metabolism* 10.1038/s42255-024-01053-4, published online 31 May 2024.

This article was originally published under standard Springer nature license (© The Author(s), under exclusive licence to Springer Nature Limited). It is now available as an open-access paper under a Creative Commons Attribution 4.0 International license, © The Author(s). The error has been corrected in the HTML and PDF versions of the article.

