# Supplementary material for: PNPO–PLP axis senses prolonged hypoxia in macrophages by regulating lysosomal activity
Source: Nat Metab. 2024 May 31;6(6):1108–27. doi: 10.1038/s42255-024-01053-4 (PMC11599045; doi:10.1038/s42255-024-01053-4)
Supplement: Supplementary file 1 — Reporting Summary [file 42255_2024_1053_MOESM1_ESM.pdf]

nature portfolio

Corresponding author(s): Hiroki Sekine & Hozumi Motohashi

Last updated by author(s): April 7, 2024

Reporting Summary

Nature Portfolio wishes to improve the reproducibility of the work that we publish. This form provides structure for consistency and transparency in reporting. For further information on Nature Portfolio policies, see our Editorial Policies and the Editorial Policy Checklist.

Statistics

For all statistical analyses, confirm that the following items are present in the figure legend, table legend, main text, or Methods section.

n/a	Confirmed	
☐	☒	The exact sample size (*n*) for each experimental group/condition, given as a discrete number and unit of measurement
☐	☒	A statement on whether measurements were taken from distinct samples or whether the same sample was measured repeatedly
☐	☒	The statistical test(s) used AND whether they are one- or two-sided<br>*Only common tests should be described solely by name; describe more complex techniques in the Methods section.*
☒	☐	A description of all covariates tested
☐	☒	A description of any assumptions or corrections, such as tests of normality and adjustment for multiple comparisons
☐	☒	A full description of the statistical parameters including central tendency (e.g. means) or other basic estimates (e.g. regression coefficient) AND variation (e.g. standard deviation) or associated estimates of uncertainty (e.g. confidence intervals)
☐	☒	For null hypothesis testing, the test statistic (e.g. F, t, r) with confidence intervals, effect sizes, degrees of freedom and P value noted<br>*Give P values as exact values whenever suitable.*
☒	☐	For Bayesian analysis, information on the choice of priors and Markov chain Monte Carlo settings
☒	☐	For hierarchical and complex designs, identification of the appropriate level for tests and full reporting of outcomes
☐	☒	Estimates of effect sizes (e.g. Cohen's d, Pearson's r), indicating how they were calculated

Our web collection on statistics for biologists contains articles on many of the points above.

Software and code

Policy information about availability of computer code

Data collection	Next generation sequencers: HiSeq 2500 sequencing system, HiSeq X ten system, Mass spectometry: Q Exactive (IC/HRMS/MS and PFPP-LC/HRMS/MS)
Data analysis	FastQC version 0.11.5, Cutadapt version 1.15, STAR version 2.5.3a, Cufflinks suite version 2.2.1, Enrichr, ggplot2, pheatmap, Rstudio version 2021.09.1+372, GSEA version 4.1.0., LASX software, ImageJ, GraphPad Software Prisom 7.

For manuscripts utilizing custom algorithms or software that are central to the research but not yet described in published literature, software must be made available to editors and reviewers. We strongly encourage code deposition in a community repository (e.g. GitHub). See the Nature Portfolio guidelines for submitting code & software for further information.

Data

Policy information about availability of data

All manuscripts must include a data availability statement. This statement should provide the following information, where applicable:

- Accession codes, unique identifiers, or web links for publicly available datasets
- A description of any restrictions on data availability
- For clinical datasets or third party data, please ensure that the statement adheres to our policy

RNA-seq data were deposited at GEO (GSE181641, GSE181642, GSE181643).
GSE181641: https://www.ncbi.nlm.nih.gov/geo/query/acc.cgi?acc=GSE181641

GSE181642: https://www.ncbi.nlm.nih.gov/geo/query/acc.cgi?acc=GSE181642
GSE181643: https://www.ncbi.nlm.nih.gov/geo/query/acc.cgi?acc=GSE181643
Metabolome data are shown in Supplementary Table 3.
mm9 reference genome: https://www.gencodegenes.org/mouse/release_M1.html

Research involving human participants, their data, or biological material

Policy information about studies with human participants or human data. See also policy information about sex, gender (identity/presentation), and sexual orientation and race, ethnicity and racism.

Reporting on sex and gender	Not applicable
Reporting on race, ethnicity, or other socially relevant groupings	Not applicable
Population characteristics	Not applicable
Recruitment	Not applicable
Ethics oversight	Not applicable

Note that full information on the approval of the study protocol must also be provided in the manuscript.

Field-specific reporting

Please select the one below that is the best fit for your research. If you are not sure, read the appropriate sections before making your selection.

☒ Life sciences ☐ Behavioural & social sciences ☐ Ecological, evolutionary & environmental sciences

For a reference copy of the document with all sections, see nature.com/documents/nr-reporting-summary-flat.pdf

Life sciences study design

All studies must disclose on these points even when the disclosure is negative.

Sample size	In this study, no sample size calculation was performed.<br>For RNA-seq and metabolome analyses, biological duplicates were used for data acquisition. We consider this was sufficient because results obtained form each analysis was verified in alternative experiments. RT-PCR for RNA-seq and VB6 Enzymatic assay kit for metabolome analysis.<br>For RT-PCR and immunoblot analysis of macrophages and cell lines, 3-5 samples were examined. For microscopic observation, 6-7 fields per sample were examined, and independent experiments were repeated for more than 3 times. For mouse experiments, more than 3 or more mice were analyzed considering the individual variation of inbred mouse strain. These samples sizes were chosen based on previous experience and on what is common practice in the field.
Data exclusions	No data were excluded.
Replication	How often the experiments were replicated or performed independently has been described in each figure legend.
Randomization	For mouse experiment, wild-type C57BL/6N male and female mice were randomly divided into the required number of groups for each experiment.
Blinding	Histological evaluation of mouse intestine samples was blindly performed. In other experiments, there is no need to conduct blind tests because the characteristics of objective and quantitative data do not allow room for arbitrary evaluations by the experimenter.

Reporting for specific materials, systems and methods

We require information from authors about some types of materials, experimental systems and methods used in many studies. Here, indicate whether each material, system or method listed is relevant to your study. If you are not sure if a list item applies to your research, read the appropriate section before selecting a response.

Materials & experimental systems

n/a	Involved in the study	
☐	☒	Antibodies
☐	☒	Eukaryotic cell lines
☒	☐	Palaeontology and archaeology
☐	☒	Animals and other organisms
☒	☐	Clinical data
☒	☐	Dual use research of concern
☒	☐	Plants

Methods

n/a	Involved in the study	
☒	☐	ChIP-seq
☒	☐	Flow cytometry
☒	☐	MRI-based neuroimaging

Antibodies

Antibodies used

ChIP assay: anti-H3K27ac antibody (MABI0309, MAB Institute), anti-H3K4me3 antibody (MABI0304, MAB Institute)
Immunoblot analysis: anti-TET2 antibody (ab124297, Abcam), KDM5a antibody (ab70892, Abcam), anti-KDM6a antibody (33510S, Cell Signaling), anti-PHD2 antibody (NB100-2219, Novus), anti-Lamp1 antibody (ab25245, Abcam), anti-PNPO antibody (15552-1-AP, Proteintech), anti-Tubulin antibody (T9026, Sigma), anti-HIF-2[illegible] (ab109616, Abcam), anti-p70 S6K (#2708, Cell Signaling Technology), anti-phospho-p70 S6K (#9234, Cell Signaling Technology), anti-pimonidazole (Pab2627, Hypoxyprobe, Inc.).
Immunohistochemistry: anti-pimonidazole (Pab2627, Hypoxyprobe, Inc.).

Validation

https://www.genetex.com/Product/Detail/Histone-H3K27ac-Acetyl-Lys27-antibody-MABI0309/GTX50903
https://www.abcam.com/tet2-antibody-ab124297.html
https://www.abcam.com/kdm5a--jarid1a--rbbp2-antibody-ab70892.html
https://www.cellsignal.jp/products/primary-antibodies/utx-d3q1i-rabbit-mab/33510
https://www.citeab.com/antibodies/408081-nb100-2219h-egln1-phd2-antibody-hrp
https://www.abcam.com/lamp1-antibody-1d4b-ab25245.html
https://www.ptglab.com/products/PNPO-Antibody-15552-1-AP.htm
https://www.sigmaaldrich.com/JP/ja/product/sigma/t9026
chrome-extension://efaidnbmnnnibpcajpcglclefindmkaj/https://site.hypoxyprobe.com/knowledge-center-articles/HP-PAb2627-Antibody-Insert.pdf
https://www.citeab.com/antibodies/763559-ab109616-anti-hif-2-alpha-antibody
https://www.cellsignal.jp/products/primary-antibodies/p70-s6-kinase-49d7-rabbit-mab/2708
https://www.cellsignal.jp/products/primary-antibodies/phospho-p70-s6-kinase-thr389-108d2-rabbit-mab/9234

Eukaryotic cell lines

Policy information about cell lines and Sex and Gender in Research

Cell line source(s)

U937--human lung (lymphoblast), HeLa--human cervical cancer.

Authentication

U937 was not authenticated. HeLa was purchased from RIKEN BRC but not authenticated by ourselves.

Mycoplasma contamination

U937 and HeLa were verified as negative for mycoplasma infection.

Commonly misidentified lines
(See ICLAC register)

Commonly misidentified cell lines were not used in this study.

Animals and other research organisms

Policy information about studies involving animals; ARRIVE guidelines recommended for reporting animal research, and Sex and Gender in Research

Laboratory animals

4-5 month-old ISAM (Yamazaki et al., Nat Commun 2013; Souma et al., J Am Soc Nephrol 2016) and their control mice for DSS colitis
2-3 month-old C57BL/6N mice for hypoxic exposure
2-4 month-old wild-type mice, Hif1aF/F:Tie2-Cre mice, VhlF/F:Lys-Cre mice in C57BL/6 background for culturing bone marrow-derived macrophages
2-4 month-old Hif2aF/F:ROSA-CreERT2 mice and PnpoF/F:ROSA-CreERT2 mice in mixed background for culturing bone marrow-derived macrophages
These mice were housed in a specific pathogen-free facility and maintained under constant temperature (24 ºC), humidity (40%), and a light/dark cycle with 12 hours of light and 12 hours of darkness, with food and water provided ad libitum.

Wild animals

Not used.

Reporting on sex

Male ISAM were used for DSS colitis experiment because males exhibit systemic hypoxia with less individual variation than females. Male and female mice were used for hypoxia exposure experiment.

Field-collected samples

No field collected samples were used in this study.

Ethics oversight	The Standards for Human Care and Use of Laboratory Animals of Tohoku University<br>The Guideline at Jichi Medical University upon approval of the Use and Care of Experimental Animals Committee of Jichi Medical University<br>The Guidelines for Proper Conduct of Animal Experiments by the Ministry of Education, Culture, Sports, Science, and Technology of Japan

Note that full information on the approval of the study protocol must also be provided in the manuscript.

Plants

Seed stocks	Not applicable.
Novel plant genotypes	Not applicable.
Authentication	Not applicable.
