# Supplementary material for: PNPO–PLP axis senses prolonged hypoxia in macrophages by regulating lysosomal activity
Source: Nat Metab. 2024 May 31;6(6):1108–27. doi: 10.1038/s42255-024-01053-4 (PMC11599045; doi:10.1038/s42255-024-01053-4)

Figure 3b

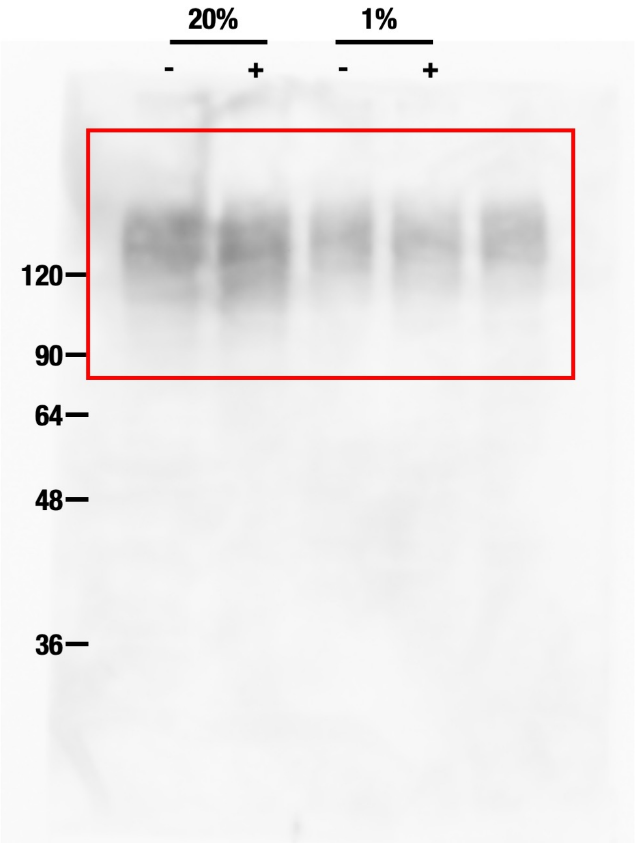

Lamp1

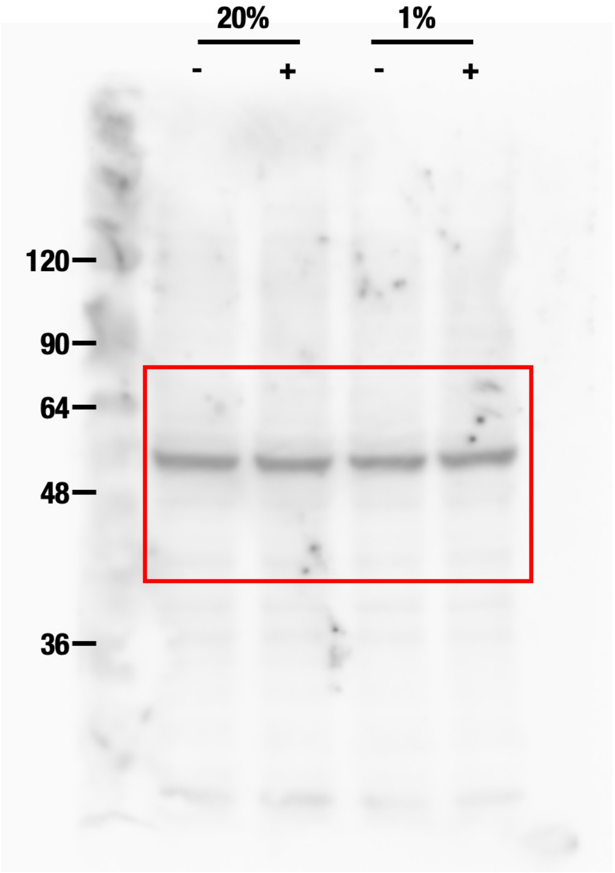

Tubulin

Figure 5a

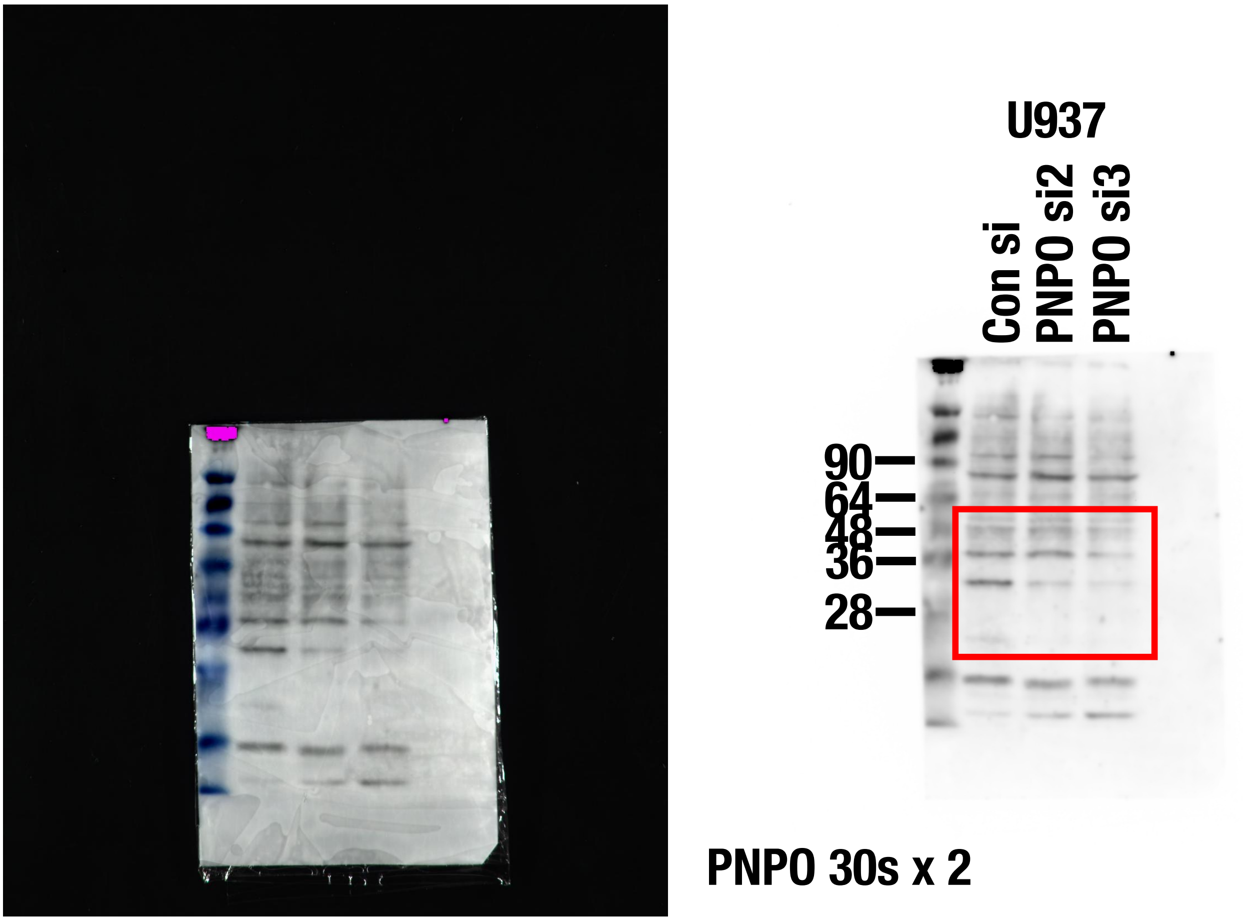

PNPO 30s x 2

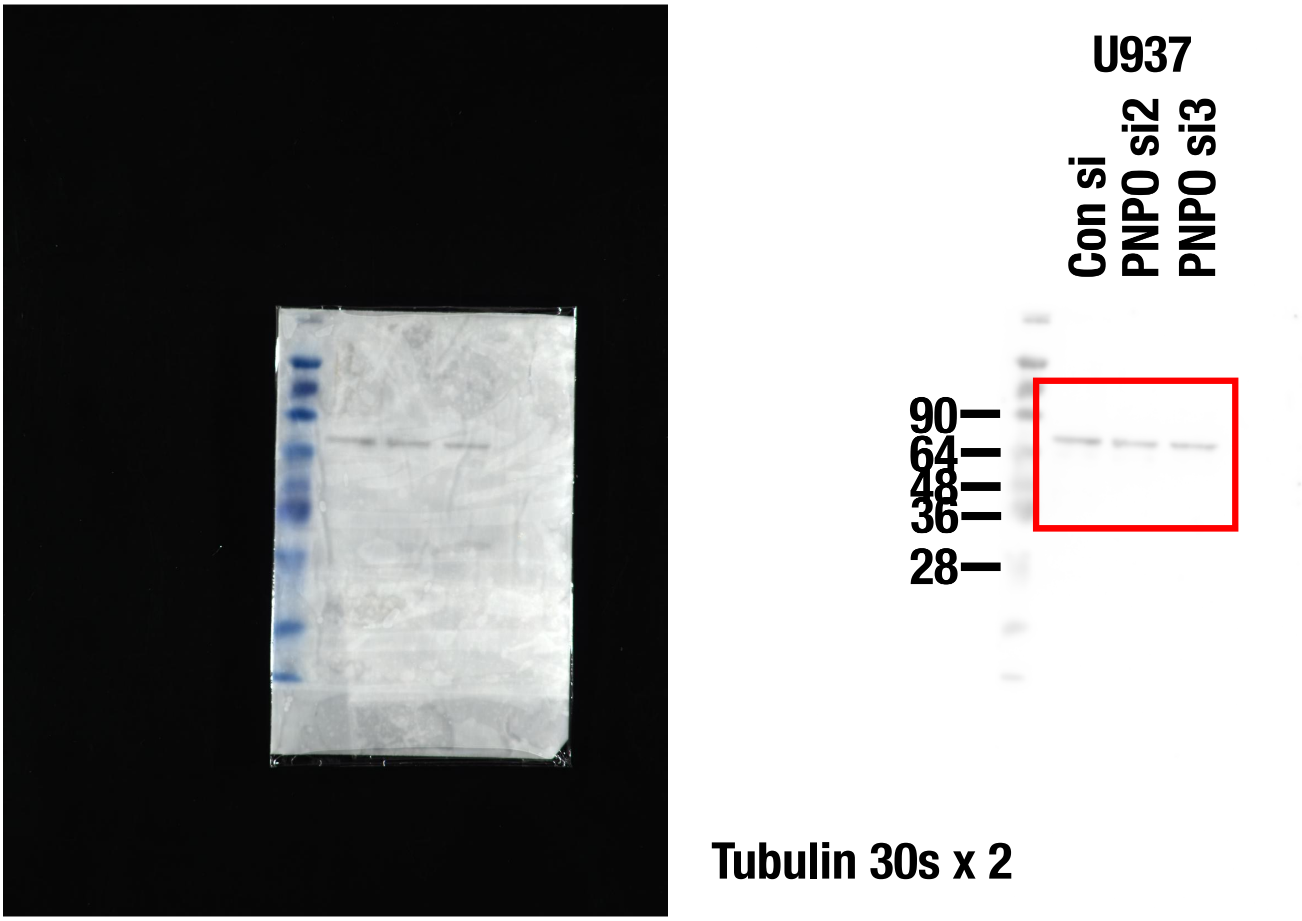

Tubulin 30s x 2

Figure 5c

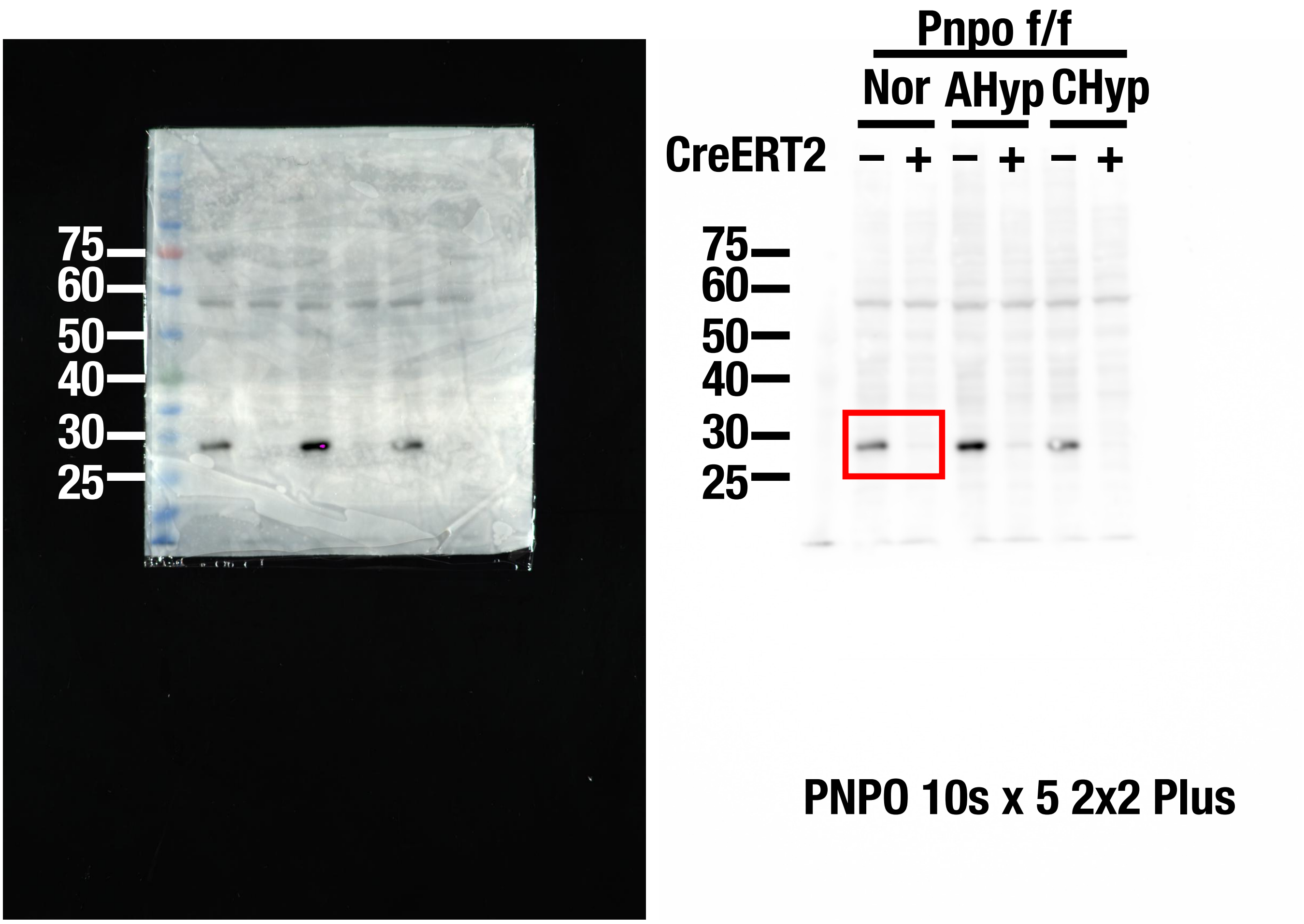

75
60
50
40
30
25
Pnpo f/f
Nor
AHyp
CHyp
CreERT2
– + – + – +
75
60
50
40
30
25
PNPO 10s x 5 2x2 Plus

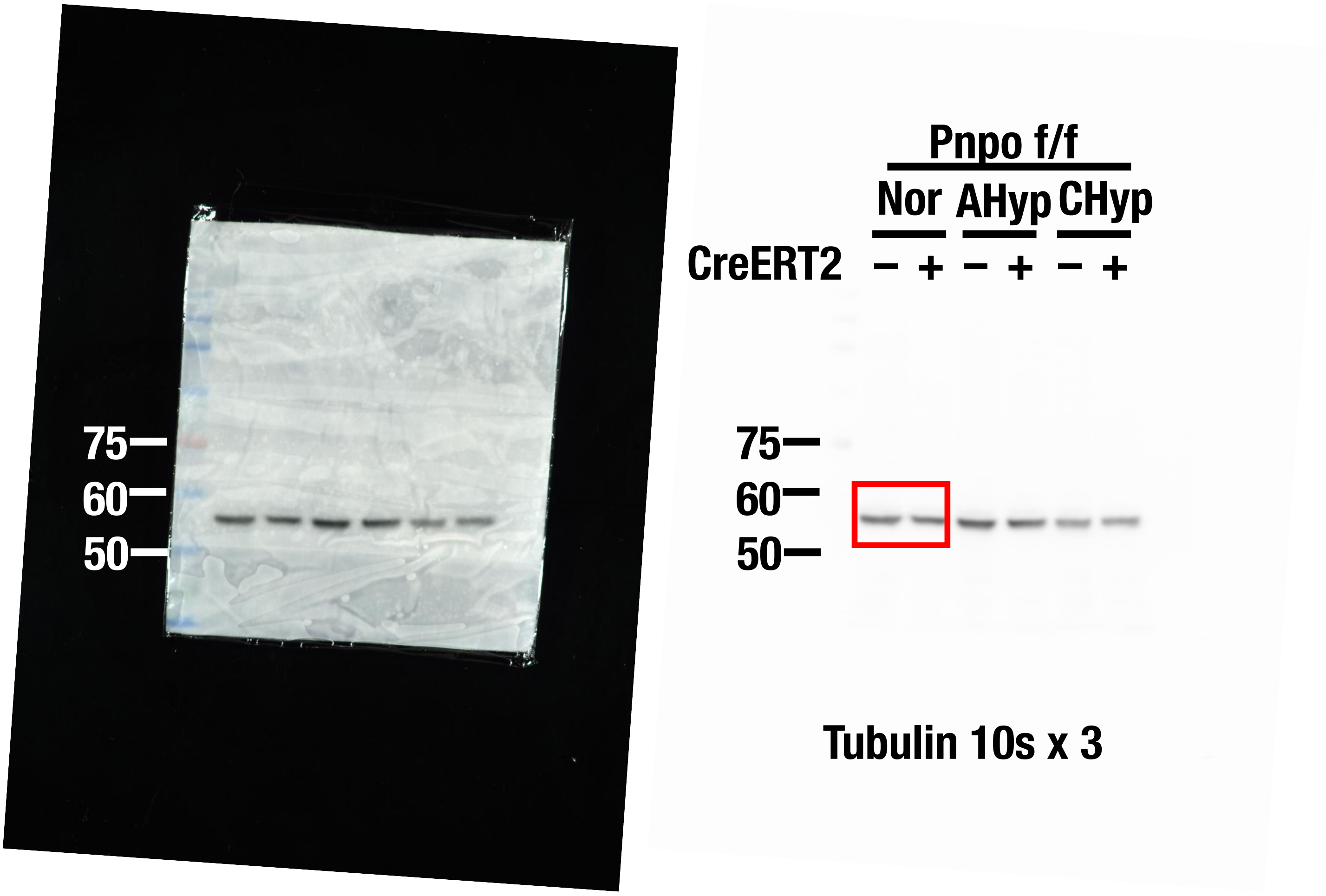

75
60
50
Pnpo f/f
Nor
AHyp
CHyp
CreERT2
– + – + – +
75
60
50
Tubulin 10s x 3

Figure 6c

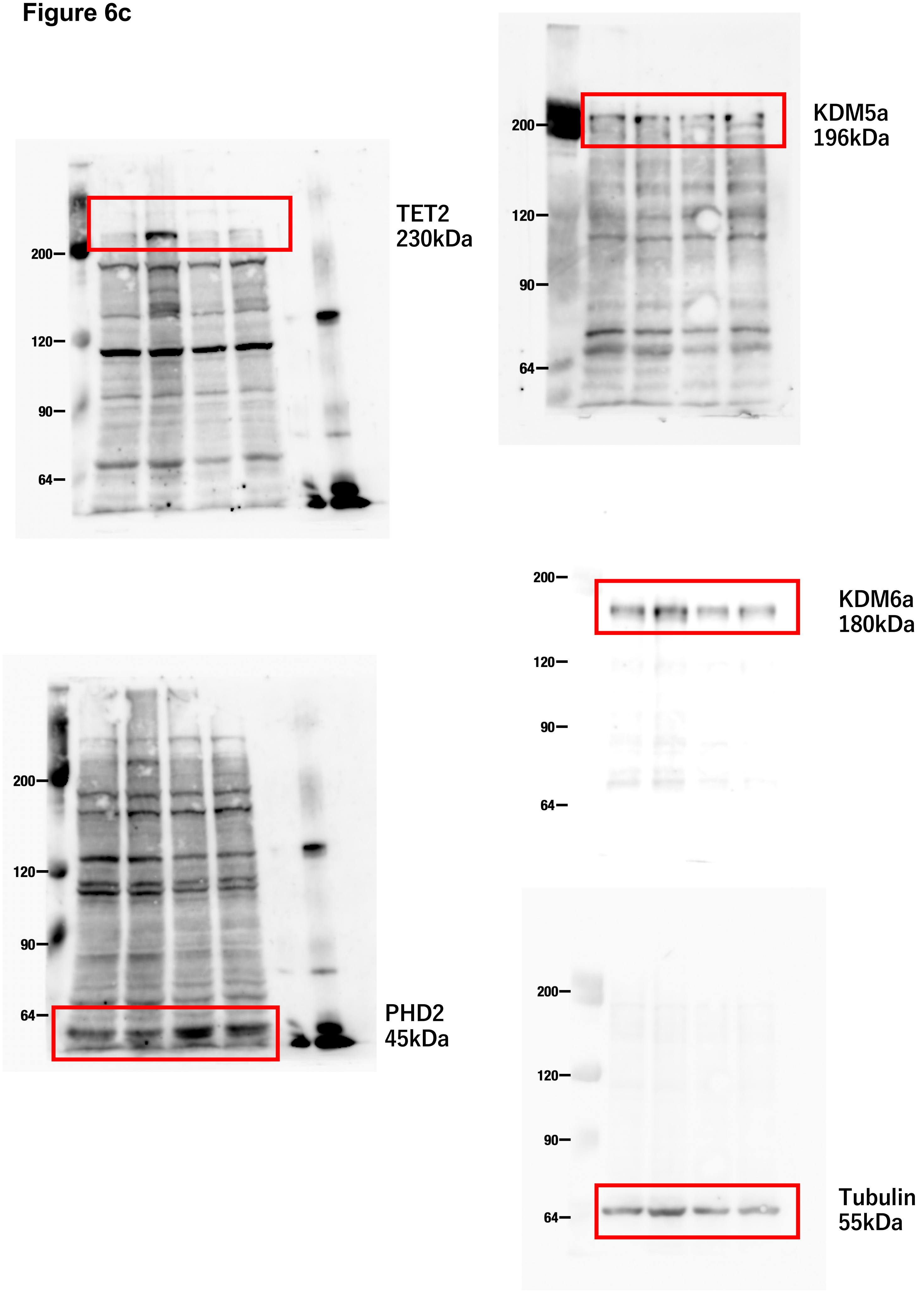

TET2
230kDa
200
120
90
64
KDM5a
196kDa
200
120
90
64
KDM6a
180kDa
200
120
90
64
PHD2
45kDa
200
120
90
64
Tubulin
55kDa
200
120
90
64

Figure 6g

TET2

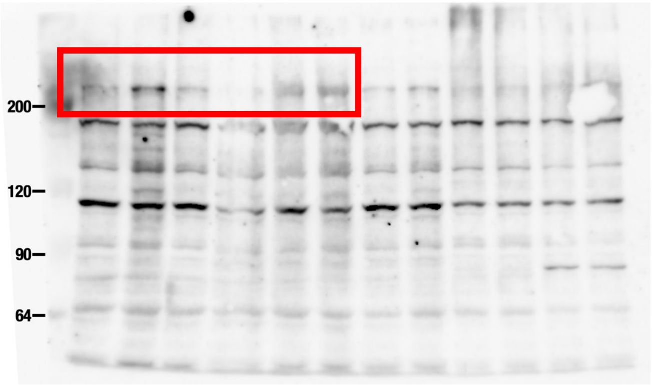

Kdm5a

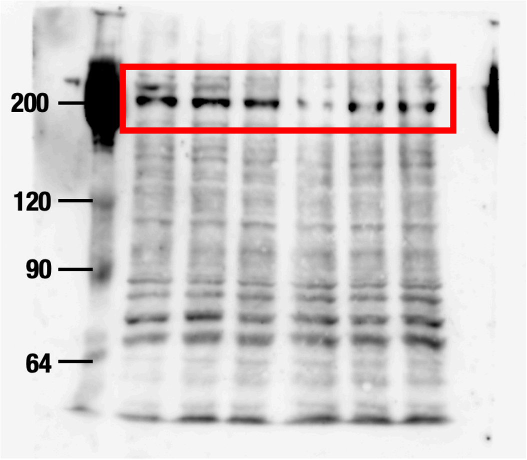

KDM6a

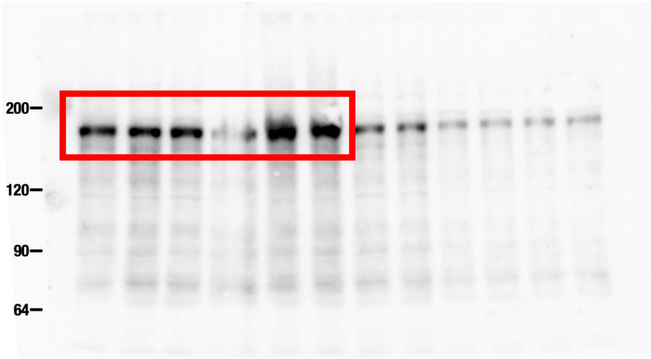

PHD2

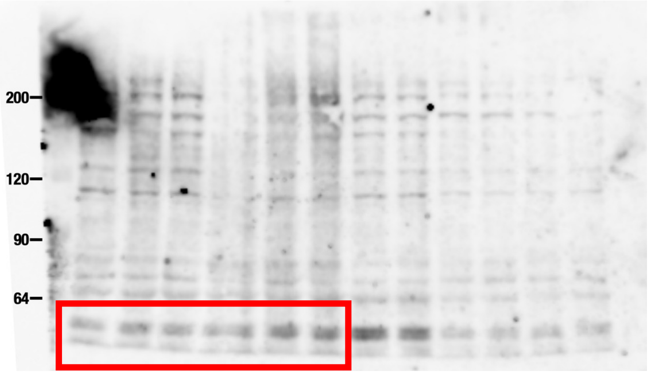

Tubulin

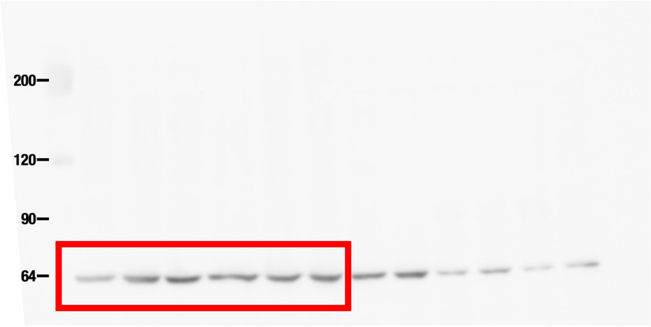

Figure 7b

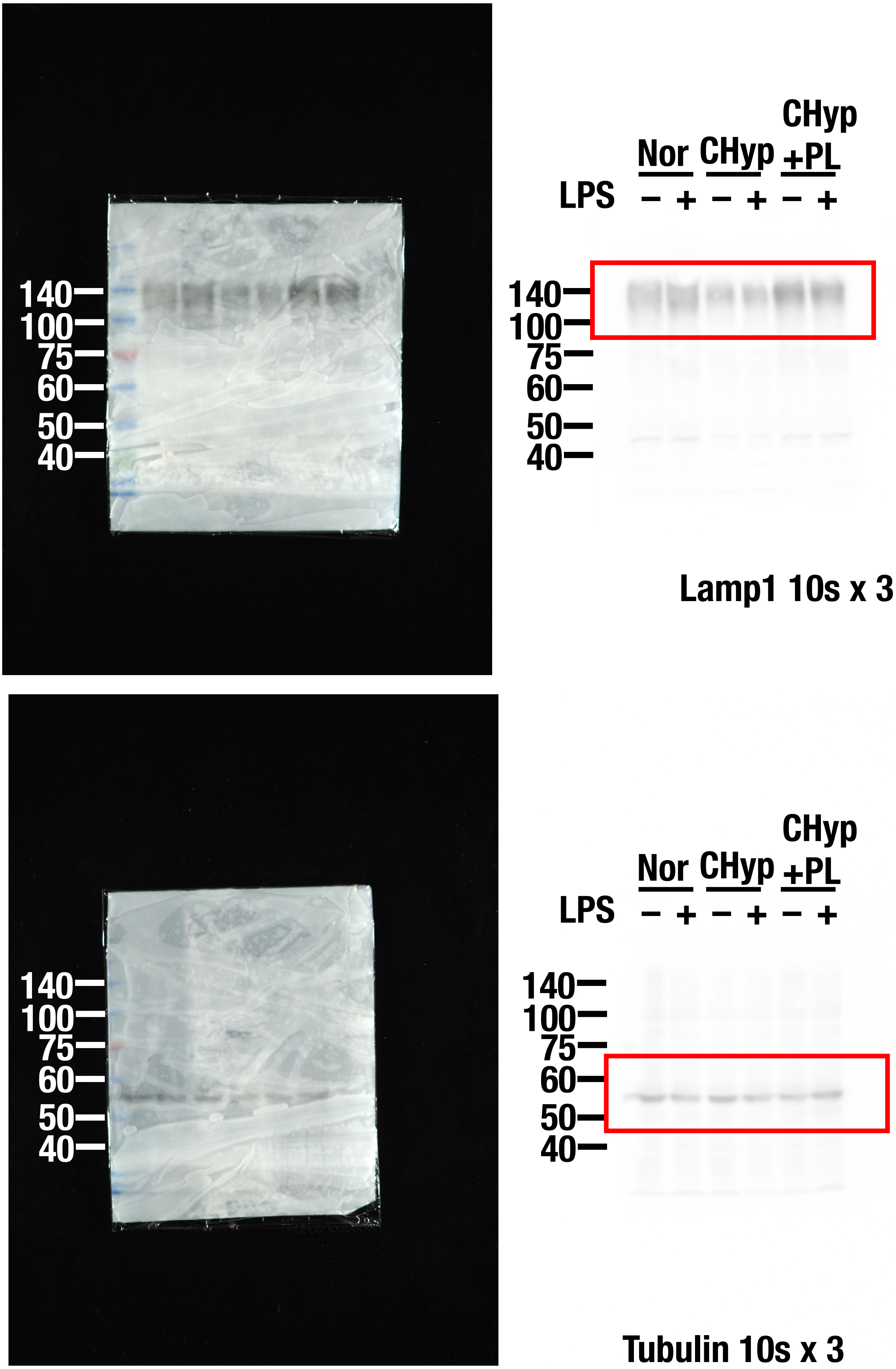

140
100
75
60
50
40
CHyp
Nor
CHyp
+PL
LPS
− + − + − +
Lamp1 10s x 3
Tubulin 10s x 3

Figure 7d

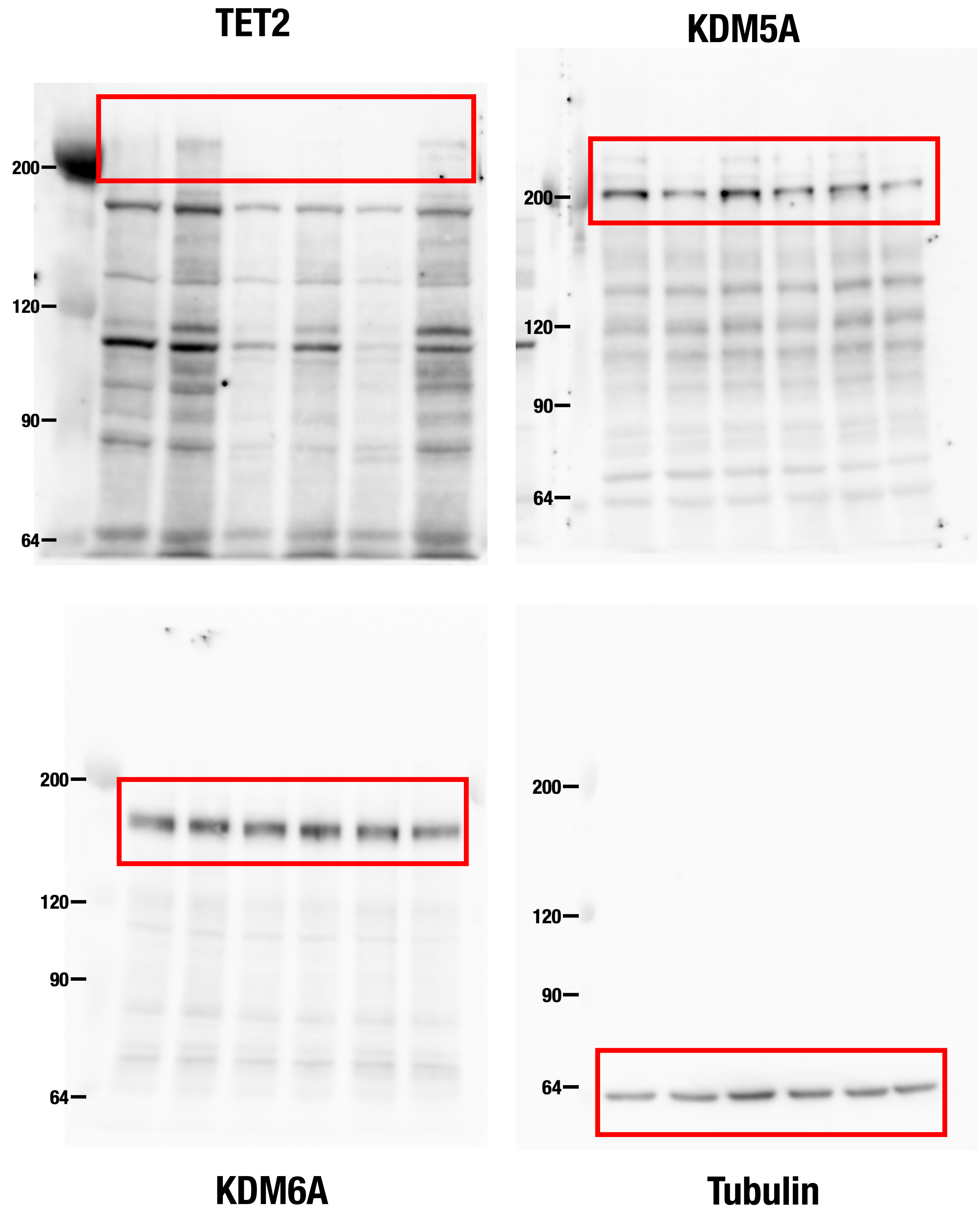
TET2
KDM5A
200
120
90
64
200
120
90
64
200
120
90
64
200
120
90
64
KDM6A
Tubulin

Extended Data Fig. 1b
(data shown in Extended Data Fig. 1b are not cropped.)

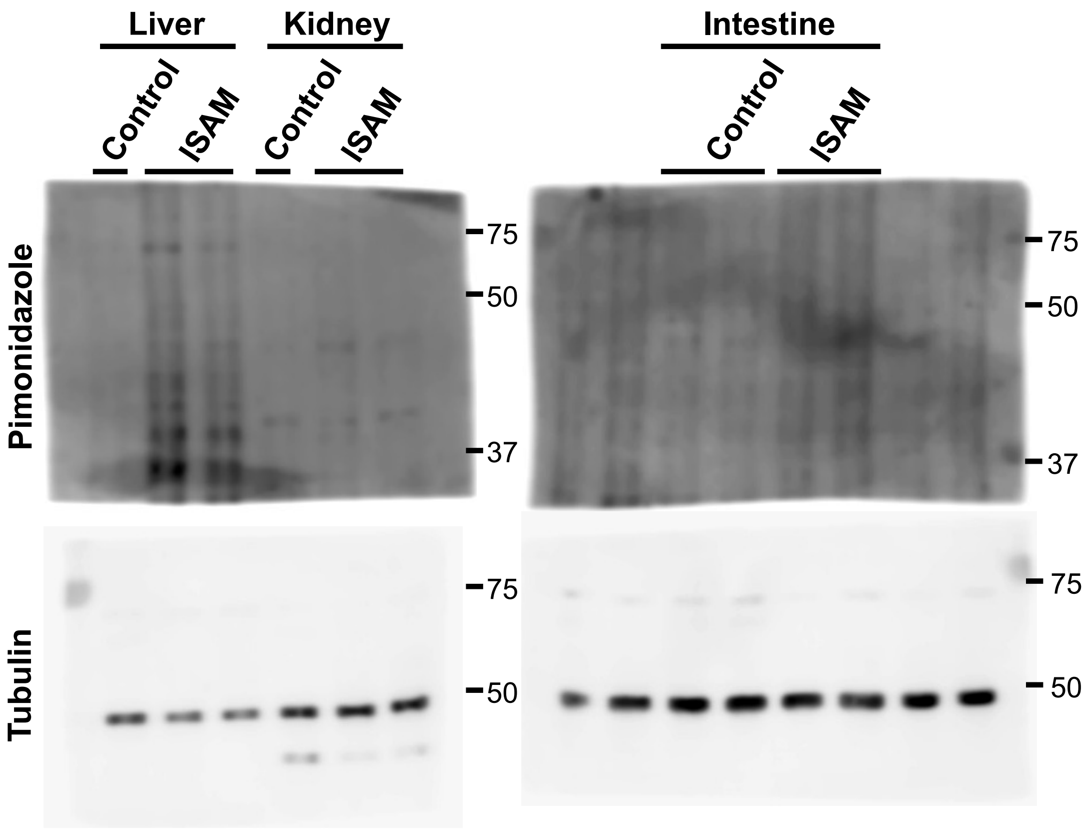

Extended Data Fig. 2e

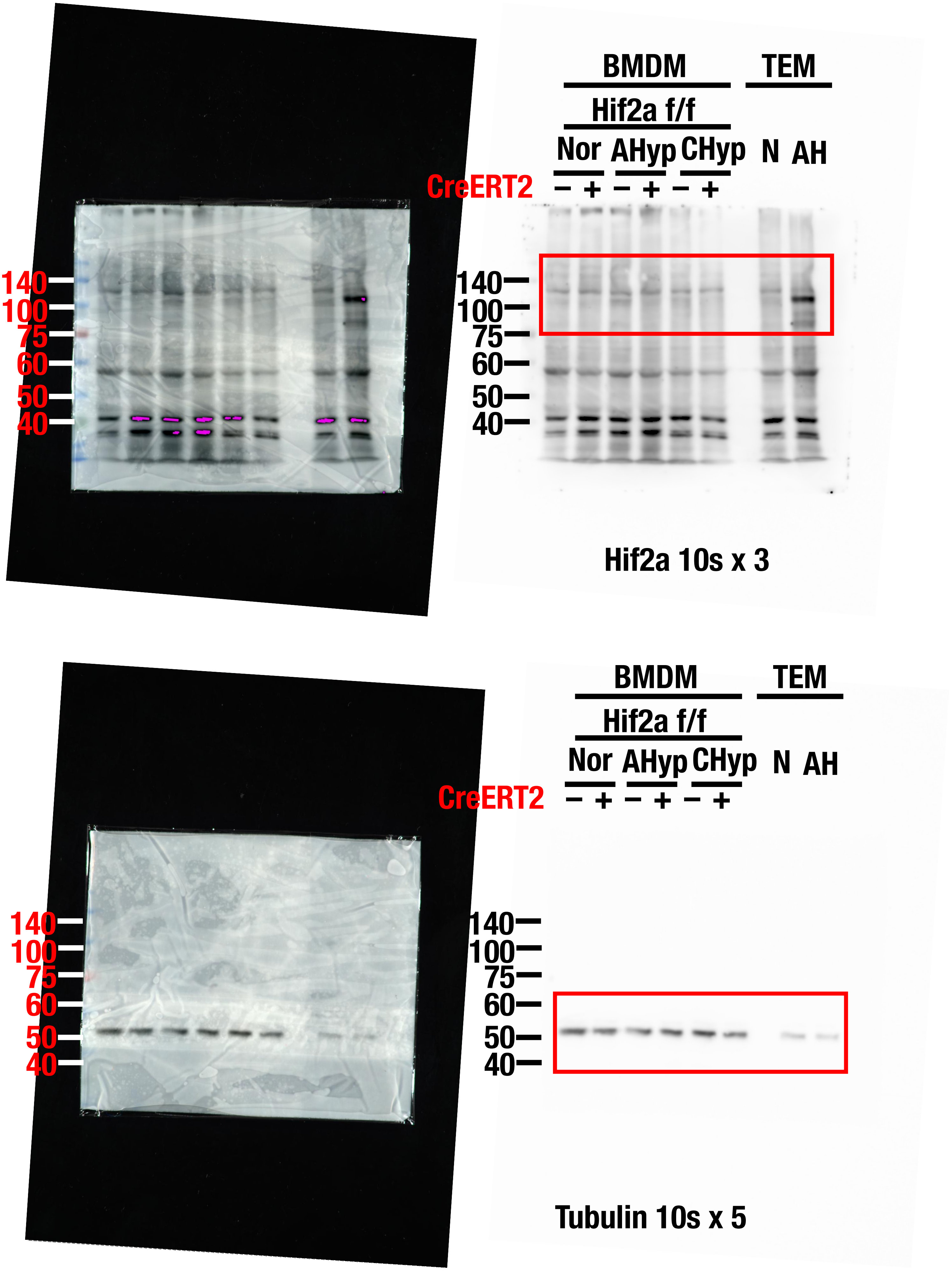

Extended Data Fig. 2g

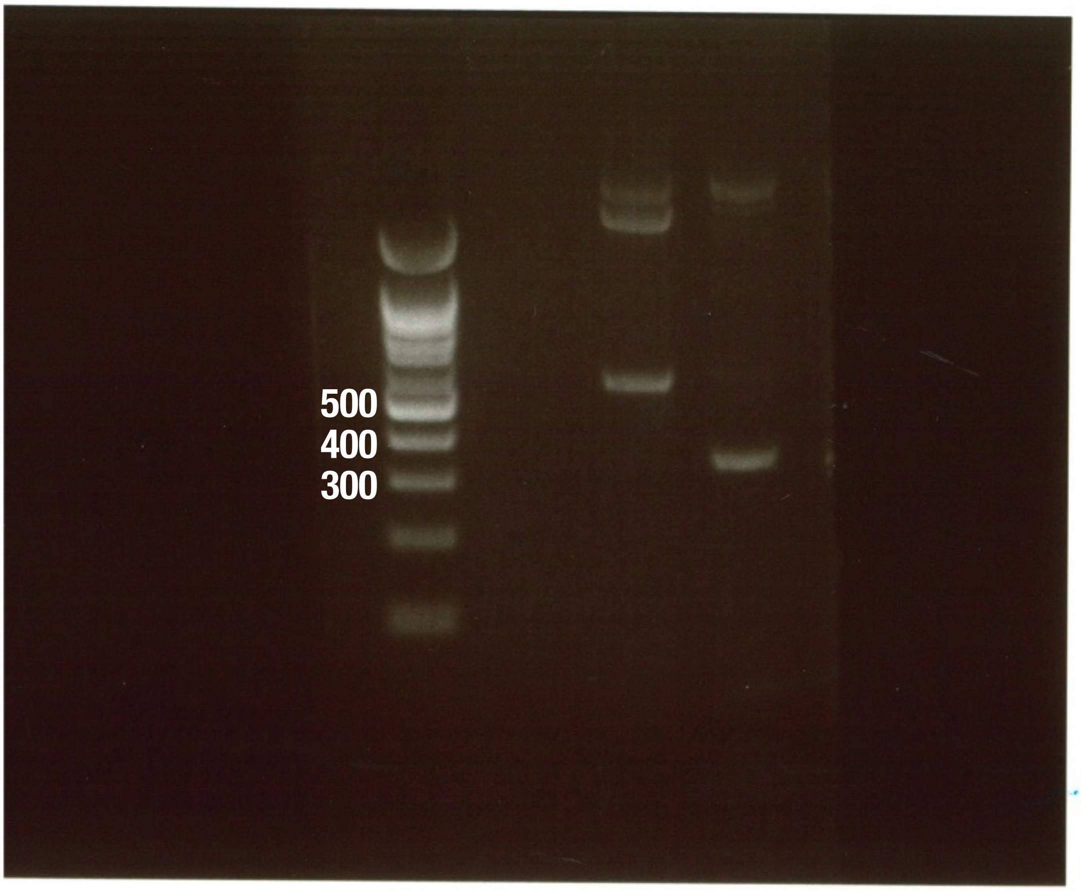

Extended Data Fig. 5a

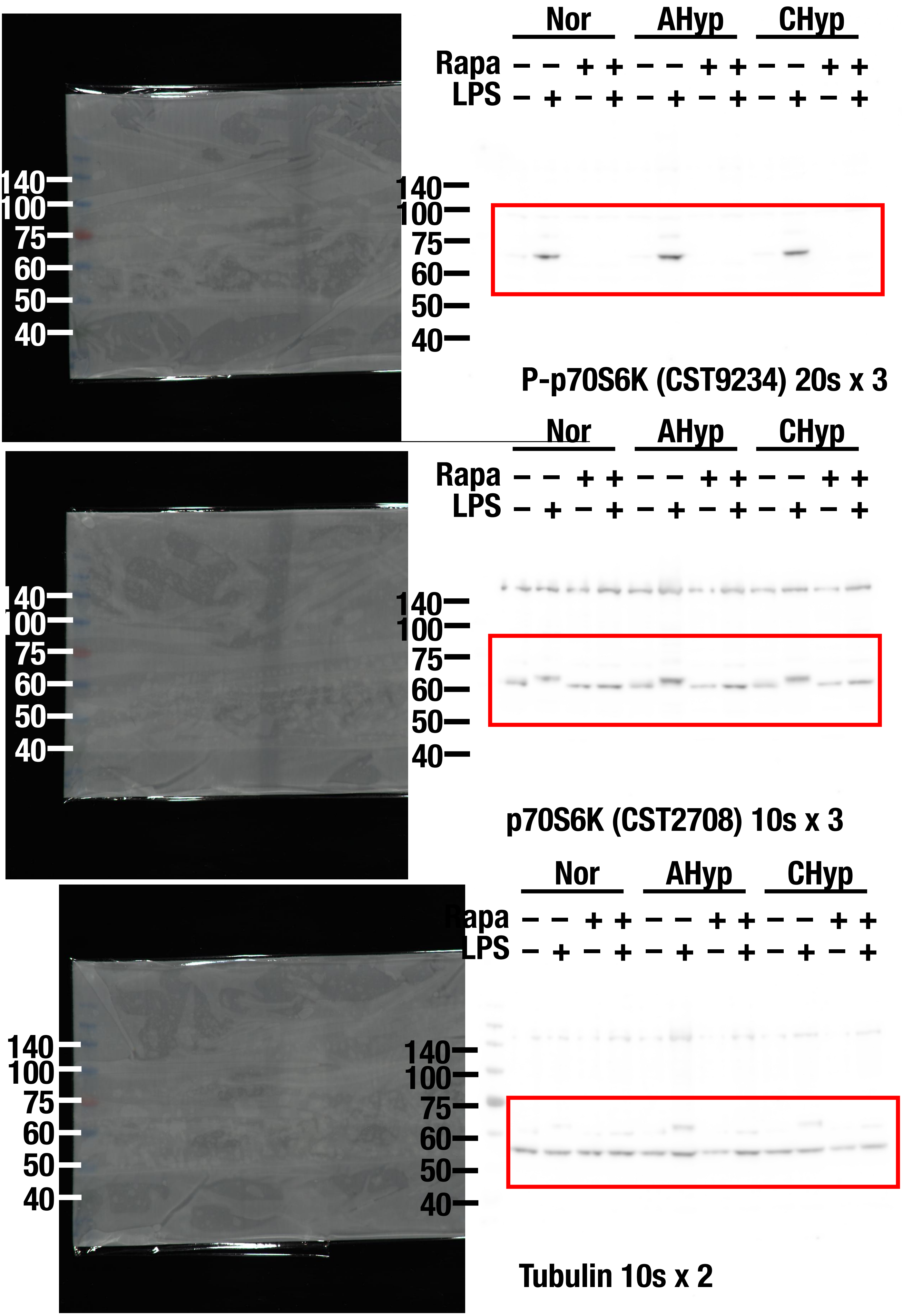

Supplement: Supplementary file 22 — Unprocessed blots and gels for Figs. 3, 5–7 and Extended Data Figs. 1, 2 and 5. [file 42255_2024_1053_MOESM22_ESM.pdf]
